# Applications of biaryl cyclization in the synthesis of cyclic enkephalin analogs with a highly restricted flexibility

**DOI:** 10.1007/s00726-023-03371-5

**Published:** 2024-03-01

**Authors:** Maria Różanowska, Gabriela Szczupaj, Michał Nowakowski, Priyadharshni Rajagopal, Piotr F. J. Lipiński, Joanna Matalińska, Aleksandra Misicka, Marek Lisowski, Łukasz Jaremko, Mariusz Jaremko

**Affiliations:** 1 Faculty of Chemistry, University of Wrocław, Wrocław, Poland; 2 Faculty of Chemistry, Biological and Chemical Research Centre, University of Warsaw, Warsaw, Poland; 3 Bioscience Program, Division of Biological and Environmental Sciences and Engineering (BESE), King Abdullah University of Science and Technology (KAUST), Thuwal, Saudi Arabia; 4 Department of Neuropeptides, Mossakowski Medical Research Institute, Polish Academy of Sciences, Warsaw, Poland; 5 Faculty of Chemistry, University of Warsaw, Warsaw, Poland

**Keywords:** Cyclization, Biaryl bridges in peptides, Miyaura–Suzuki reaction, NMR, CD, Molecular docking

## Abstract

A series of 10 cyclic, biaryl analogs of enkephalin, with Tyr or Phe residues at positions 1 and 4, were synthesized according to the Miyaura borylation and Suzuki coupling methodology. Biaryl bridges formed by side chains of the two aromatic amino acid residues are of the *meta*–*meta*, *meta*–*para*, *para*–*meta*, and *para*–*para* configuration. Conformational properties of the peptides were studied by CD and NMR. CD studies allowed only to compare conformations of individual peptides while NMR investigations followed by XPLOR calculations provided detailed information on their conformation. Reliability of the XPLOR calculations was confirmed by quantum chemical ones performed for one of the analogs. No intramolecular hydrogen bonds were found in all the peptides. They are folded and adopt the type IV β-turn conformation. Due to a large steric strain, the aromatic carbon atoms forming the biaryl bond are distinctly pyramidalized. Seven of the peptides were tested in vitro for their affinity for the µ-opioid receptor.

**Supplementary Information:**

The online version contains supplementary material available at 10.1007/s00726-023-03371-5.

## Introduction

The cyclization of peptides is an important issue in peptide chemistry. It has manifold goals. It is very helpful in conformational studies, especially in the case of short peptides since it limits their flexibility and enables determination of the dominant conformation. Still greater importance cyclization has in the case of biologically active peptides. Cyclic peptides are more resistant to enzymatic degradation and thus their stability and life span are increased (Bechtler and Lamers 2021; Frost et al. 2016). They show also enhanced membrane permeability (Hayes et al. 2021). These features improve significantly the pharmacological properties of such compounds and their therapeutic potential.

Peptides can be cyclized by various ways among which side chain-to-side chain cyclization is quite often used. A special method of such a cyclization in peptides is a direct connection of two aromatic rings in the side chains of aromatic amino acid residues, resulting in the biaryl bridge. Biaryl bridges in peptides can be obtained by various methods. One of them is catalytic oxidative cross-coupling reaction (Ben-Lulu et al. 2020). A series of biaryl-bridged (via two tyrosine residues) peptides were synthesized by this method with the use of iron catalyst and urea hydrogen peroxide as an oxidant, and later cyclized by lactamization. An important factor in this approach is the presence of an activating *tert*-butyl group at the *ortho* positions in the aromatic rings of both tyrosine residues. Another method of synthesis of cyclic biaryl peptides is cyclization of peptides with iodinated aromatic ring at the C-terminus and the N-terminal benzamide group, catalyzed by palladium (Bai et al. 2019).

A very useful method of synthesis of biaryl bridges in peptides is Miyaura borylation–Suzuki coupling (Miyaura and Suzuki 1995). Initially, it was a reaction between an organoboronic acid and a halide, catalyzed by palladium. In the case of biaryls, aryl iodides or bromides were used since aryl chlorides are quite inert to oxidative addition. Apart from organoboronic acids, many other boron-based compounds have been introduced into Miyaura–Suzuki reaction (Willemse et al. 2017). They are more stable and react more productively. The advantage of this reaction is that it proceeds under relatively mild conditions and with various organoboronic compounds as substrates. Synthesis of cyclic biaryl-bridged peptides by the Miyaura–Suzuki method requires then the presence of two suitably derivatized aromatic amino acid residues in their sequence—an arylboronic derivative and an aryl halide. Due to its versatility and reaction conditions, this method has found a wide application to the synthesis of cyclic biaryl peptides. It has been used for the first time for synthesis of the biphenomycin model (Carbonnelle and Zhu 2000) and soon later for synthesis of the functionalized macrocyclic core of proteasome inhibitors TMC-95A and B (Kaiser et al. 2002; Lin and Danishefsky 2001, 2002). Since then many cyclic biaryl peptides have been synthesized according to the Miyaura–Suzuki approach. They contain mainly Phe–Phe, Phe–Tyr, and Tyr–Tyr linkages (Afonso et al. 2011, 2012; Carbonnelle and Zhu 2000; García-Pindado et al. 2017; Han et al. 2020; Kaiser et al. 2002; Lin and Danishefsky 2001, 2002; Mendive-Tapia et al. 2015; Meyer et al. 2012; Ng-Choi et al. 2019b). Syntheses of cyclic biaryl peptides with the Phg–Phe, Tyr–Trp, His–Tyr, Trp–Phe, and Trp–Trp bridges have been also reported (García-Pindado et al. 2018; Gruß et al. 2022; Gruß and Sewald 2020; Han et al. 2020; Kaiser et al. 2002; Kemker et al. 2019; Lin and Danishefsky 2001, 2002; Mendive-Tapia et al. 2015; Ng-Choi et al. 2019a, 2020).

In this paper, we describe the synthesis and conformational studies on cyclic biaryl analogs of enkephalins (Hughes et al. 1975). Enkephalins are natural linear pentapeptides isolated from brain extracts. Their sequences are Tyr–Gly–Gly–Phe–Leu and Tyr–Gly–Gly–Phe–Met. They are endogenous peptides which belong to the opioid peptides class and exhibit morphine-like properties (Beluzzi et al. 1976). During the structure–activity studies on the enkephalins, many cyclic analogs of the peptides have been synthesized (Remesic et al. 2016). Cyclizations have been performed by various ways. Cyclic enkephalin analogs were obtained most often by bridging the peptide chain at positions 2 and 5, e.g., with the use of penicillamines (Mosberg et al. 1983; Hruby et al. 1997), methylamine (Shreder et al. 1998), carbonyl group (Pawlak et al. 2001), and lanthionine (Rew et al. 2002). By contrast, to the best of our knowledge, the aromatic residual side chains of enkephalins were used only once for cyclization (Siemion et al. 1981). However, it may be a promising way in the search for potent enkephalin analogs since it was found then that azo-enkephalin, in which the Tyr^1^ and Phe^4^ aromatic rings were linked with each other by the azo bridge, is very active in in vivo tests.

**Fig. 1:**
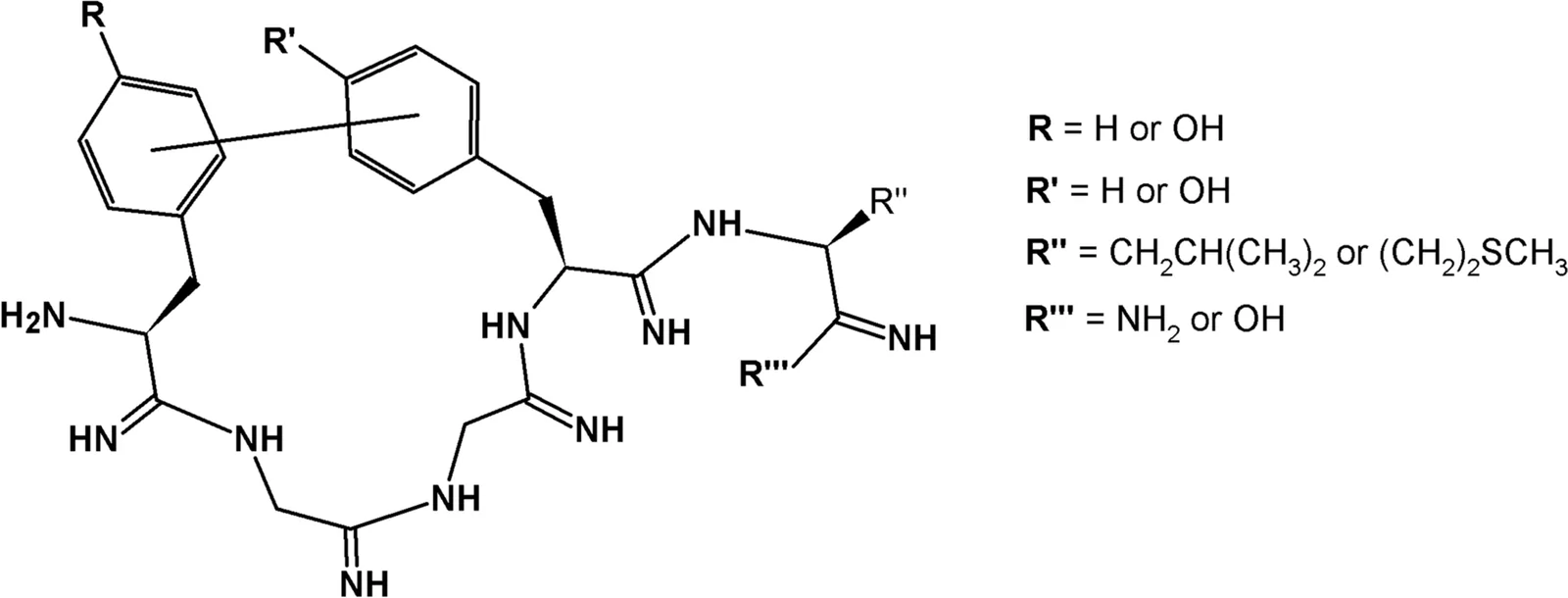
Structures of the synthesized biaryl cyclic enkephalin analogs

In the analogs presented here, the aromatic rings of the parent or substituted residues at positions 1 and 4 are situated in a very close proximity, i.e., they are connected directly with each other at different positions of the rings. The configurations of biaryl bridges obtained are *meta*–*meta*, *meta*–*para*, *para*–*meta*, and *para*–*para*. The peptides were synthesized by the Miyaura–Suzuki coupling methodology (Miyaura and Suzuki 1995). Their general structure and a list of synthesized peptides are presented below in Fig. 1 and Table 1, respectively.

**Table 1:** List of cyclic biaryl peptides synthesized

Peptides	Short names
H-(cyclo-*m,p*)-[Tyr-Gly-Gly-Phe]-Leu-NH_2_	**c-(Tyr-*m*-Phe-*p*)-L-NH**_**2**_
H-(cyclo-*m,m*)-[Tyr-Gly-Gly-Phe]-Leu-NH_2_	**c-(Tyr-*m*-Phe-*m*)-L-NH**_**2**_
H-(cyclo-*m,p*)-[Tyr-Gly-Gly-Phe]-Met-NH_2_	**c-(Tyr-*m*-Phe-*p*)-M-NH**_**2**_
H-(cyclo-*m,p*)-[Tyr-Gly-Gly-Phe]-Leu-OH	**c-(Tyr-*m*-Phe-*p*)-L-OH**
H-(cyclo*-m,m*)-[Tyr-Gly-Gly-Tyr]-Leu-NH_2_	**c-(Tyr-*m*-Tyr-*m*)-L-NH**_**2**_
H-(cyclo*-p,m*)-[Phe-Gly-Gly-Tyr]-Leu-NH_2_	**c-(Phe-*p*-Tyr-*m*)-L-NH**_**2**_
H-(cyclo-*p,p*)-[Phe-Gly-Gly-Phe]-Leu-NH_2_	**c-(Phe-*p*-Phe-*p*)-L-NH**_**2**_
H-(cyclo-*m,p*)-[Phe-Gly-Gly-Phe]-Leu-NH_2_	**c-(Phe-*m*-Phe-*p*)-L-NH**_**2**_
H-(cyclo-*m,m*)-[Phe-Gly-Gly-Phe]-Leu-NH_2_	**c-(Phe-*m*-Phe-*m*)-L-NH**_**2**_
H-(cyclo-*p,m*)-[Phe-Gly-Gly-Phe]-Leu-NH_2_	**c-(Phe-*p*-Phe-*m*)-L-NH**_**2**_

The Suzuki–Miyaura approach has already been used for synthesis of biaryl cyclic peptides with three to eight residues and with the *para–para* and *meta*–*para* configurations of the biaryl bridge (Afonso et al. 2011). In another study, biaryl cyclic tri-, tetra-, and pentapeptides have been synthesized with three different configurations: *meta*–*meta*, *meta*–*ortho*, and *ortho*–*meta* (Meyer et al. 2012). Planned CD and NMR studies on those peptides in buffered aqueous solution were not possible due to their insufficient solubility in such a medium.

The main goal of the present study was determination of spectroscopic properties of the obtained peptides and determination of their conformations. There are few papers in which X-ray structures and solution conformations of biaryl cyclic peptides are described. In 2001, the crystal structure of the 20 S proteasome:TMC-95A non-covalent complex was determined (Groll et al. 2001). TMC-95A is a cyclic biaryl peptide with the Tyr–Trp linkage which inhibits enzymatic activities of 20 S proteasome (Kohno et al. 2000). The peptide has been investigated by NMR (Kohno et al. 2000) and it has been found that its conformation in the unbound state is similar to its conformation in the complex with 20 S proteasome (Groll et al. 2001). The X-ray structure has been also determined for a cyclic biaryl peptide obtained from linear precursor PA-Phg-Gly-Leu-Phe-COOMe (PA—picolinamide), with the *ortho*–*para* Phg–Phe biaryl bridge (Han et al. 2020). NMR studies have been conducted also on a series of peptides with the Phe–Trp biaryl bridge, in which the two aromatic amino acid residues were separated by Asn–Gly–Arg, Arg–Gly–Asp, and Ser–Ala sequences and by a Val residue (Mendive-Tapia et al. 2015).

The peptides described in this paper were investigated by CD in MeOH, TFE, and water, pH 7, and NMR in H_2_O/D_2_O. NMR studies allowed a detailed characterization of their conformational properties. To the best of our knowledge, it is the first report presenting such results for cyclic biaryl peptides of the same sequences and differing in the configuration of their biaryl bridges. Since the peptides studied are analogs of enkephalins, seven of them were also tested in vitro for affinity at the μ-opioid receptor (MOR). For comparison, their linear counterparts, were tested as well. They served as reference peptides for the cyclic ones in biological studies.

## Materials and methods

### Synthesis of peptides

#### General procedure A for Fmoc SPPS Chemistry

Peptides were synthetized manually by stepwise solid-phase synthesis on a Rink Amide MBHA resin (0.68 mmol/g) and an Fmoc-Leu-Wang resin (0.65 mmol/g) according to a standard Fmoc solid-phase synthesis procedure. For the coupling of standard Fmoc-amino acids (3 eq), HATU (3 eq) in presence of HOBt (3 eq) and DIEA (6 eq) in DMF were used. For couplings using synthesized or expensive amino acids, 1.5 − 2 eq amino acid, longer reaction times (8–16 h) were used. After each coupling and deprotection step, the resin was washed 7 times with DMF. Coupling yields were monitored by quantitative ninhydrin assay (Kaiser et al. 1970). Fmoc deprotections were achieved with 25% piperidine/DMF (2 × 10 min).

#### General procedure B for Miyaura solid-phase borylation and linear precursors of peptides with the Phe residue at position 4

After completion of synthesis of Fmoc-Gly-Gly-Phe(3-I)-Leu Rink MBHA, Fmoc-Gly-Gly-Phe(4-I)-Leu Rink MBHA, Fmoc-Gly-Gly-Phe(4-I)-Met Rink MBHA, and Fmoc-Gly-Gly-Phe(4-I)-Leu Wang resin by a standard SPPS procedure, the Fmoc group was removed with 25% piperidine in DMF. The N-trityl group protection was introduced with TrCl (10 eq) and DIEA (10 eq) in DMF at room temperature for 3 h (Afonso et al. 2011). Then the resin was washed with DMF (6 × 1 min), DMF/DCM (50:50, 3 × 1 min) and DCM (3 × 1 min), *vacuum*-dried in desiccator overnight and transferred to a glass vial with PTFE Septa and magnetic stirring bar. To the vial was added bispinacolatodiboron (B_2_Pin_2_, 4 eq), Pd(dppf)Cl_2_∙CH_2_Cl_2_ (0.18 eq), dppf (0.09 eq), and KOAc (6 eq, *vacuum* dried), and degassed and saturated with nitrogen anhydrous DMSO as a solvent (2–3 ml per 100 mg of resinstored over molecular sieves, 5 times degassed in ultra-sonication bath, and saturated with nitrogen). The vial was heated in oil at bath 80 °C for 4–8 h. Then, the resin was transferred back to a propylene syringe reactor and washed with DMSO (6 × 1 min), DMSO/water (50:50, 3 × 1 min), water (6 × 1 min), water/methanol (50:50, 3 × 1 min), methanol (6 × 1 min), methanol/CH_2_Cl_2_ (50:50, 3 × 1 min), and CH_2_Cl_2_ (3 × 1 min). The boronopeptidyl resins were treated with TFA/H_2_O/DCM (0.2:1:98.8, 2 × 1 min, 1 × 20 min) and then washed with DCM (3 × 1 min), DIEA/DCM (1:19) (3 × 1 min), DMF (3 × 1 min), and DMF (3 × 1 min). Next, the boronopeptidyl resins were coupled with Boc-Tyr(^*t*^Bu,3-I)-OH, Fmoc-Phe(4-I)-OH, or Fmoc-Phe(3-I)-OH following a general procedure A. In the case of peptides with the N-terminal phenylalanine derivate, peptidyl resins were treated with 25% piperidine in DMF (2 × 10 min), washed 7 times with DMF, and treated with TrCl (10 eq) and DIEA (10 eq) in DMF at room temperature for 3 h. After completion of the reaction, the resin was washed with DMF (3 × 1 min), DMF/MeOH (50:50, 3 × 1 min), MeOH (3 × 1 min), MeOH/DCM, DCM (3 × 1 min), and *vacuum*-dried in desiccator.

#### General procedure C for Suzuki solid-phase cyclization of peptides

Resins with linear precursors of cyclic peptides containing both boron- and iodine-modified amino acids, N-protected with the Boc or trityl group, after drying in desiccator were transferred to glass vials with PTFE Septa and magnetic stirring bar. Then Pd_2_(dppf)Cl_2_∙CH_2_Cl_2_ (0.2 eq), CsF (4 eq), and degassed dioxane/water (9:1) saturated with nitrogen were added. Vials were flushed with nitrogen and sonicated under *vacuum*, saturated with nitrogen three more times, and placed in the oil bath at 80 °C. After 8–24 h, the resin was transferred to a polypropylene syringe reactor and washed with dioxane/water (6 × 1 min), water (6 × 1 min), MeOH (6 × 1 min), and DCM (6 × 1 min). In the case of peptides with the N-terminal phenylalanine derivate, the peptidyl resins were additionally treated with TFA/H_2_O/DCM (0.2:1:98.8, 2 × 1 min, 1 × 20 min) and then washed with DCM (6 × 1 min). The resulting biaryl peptidyl resin was *vacuum*-dried overnight and cleaved with TFA/H_2_O/TIS (95:2.5:2.5), treated as described in general procedure for SPPS chemistry, and purified as described in procedure Purification of peptides.

#### General procedure D for cleavage from the resin

Peptides were cleaved from the resin using tri-fluoro-acetic acid/water/TIS (95:2.5:2.5, v:v:v) for 3 h at room temperature. In the case of H-(cyclo-*m,p*)-[Tyr-Gly-Gly-Phe]-Met-NH_2_, a mixture of TFA:H_2_O:EDT:TIS (v:v:v:v 94:2,5:2,5:1) was used. Then the products were precipitated with cold diethyl ether and washed twice with cold diethyl ether (centrifuged each time). After evaporation of the ether, the samples were dissolved in 10% acetonitrile in water, lyophilized, and submitted to HPLC purification.

#### Purification of peptides

The compounds were purified on a semipreparative Varian ProStar chromatograph equipped with a Tosoh Bioscience ODS-120T column (21.5 × 300 mm, particle size: 10 µm) and a 210/254 nm dual-wavelength UV detector. For HPLC purification, solvents containing 0.1% TFA in water and 0.1% TFA in 80% acetonitrile/water and a flow rate 7 ml/min were used. Collected fractions (products confirmed by MS analysis) were lyophilized. The purity of products was confirmed by HPLC analysis with a Thermo Separation HPLC system with a UV detection (210 nm) equipped with a Vydac Protein RP C18 column (4.6 × 250 mm, particle size: 5 µm). Gradient elution of 0–80% B in 40 min was used (eluent A: 0.1% aqueous TFA in H_2_O, eluent B: 0.1% TFA in 80% acetonitrile/water), flow rate of 1 ml/min.

Details concerning reagents used and syntheses of cyclic and linear peptides are given in Supplementary Information.

### Mass spectrometry measurements

Mass spectrometric measurements (MS and MS/MS) were performed on an ESI-FT-ICR Apex-Qe 7T instrument (Bruker). The potential between the spray needle and the orifice was set to 4.5 kV. MS and MS/MS spectra an acetonitrile/water/formic acid (50:50:0.1) mixture or methanol were used. For fragmentation, the collision-induced dissociation (CID) technique was used with argon as the collision gas. The MS was calibrated with a Tunemix mixture (Bruker Daltonics).

MS and MS/MS experiment was also performed on a Shimadzu LC-IT-TOF instrument. CID fragmentation (with Argon) was used in the instrument, and the potential between the spray needle and the orifice was set to 4.5 kV. The LC systems were operated with the following mobile phases: A = 0.1% HCOOH in H_2_O and B = 0.1% HCOOH in MeCN. MS spectra were recorded without the LC column installation.

### LC–MS

LC–MS experiments were performed on a Shimadzu LC-UV-IT-TOF instrument in the positive ion mode, with electrospray ionization. The LC system was operated with the following mobile phases: A = 0.1% HCOOH in H_2_O and B = 0.1% HCOOH in MeCN in a gradient separation from 0 to 60% B/A in 15 min at a 0.1 mL/min flow rate and a 2–5 μL injection. The separations were performed on a Phenomenex C18 column (3 × 50 mm, particle size: 3.6 μm).

### CD studies

CD spectra were measured on a Jasco J-600 spectropolarimeter, at room temperature. Pathlengths of 1 and 10 mm were used for the peptide and aromatic region, respectively. Each spectrum represents the average of at least 7 scans. Concentrations of the solutions were in the range of 0.05–0.07 and 0.5–2.5 mg/ml for the peptide and aromatic region, respectively. Spectra were measured in MeOH, TFE, and water, pH 7 (0.01 M sodium phosphate buffer). The data are presented as total molar ellipticity [*θ*].

### NMR studies

All NMR spectra were recorded on 950 and 700 MHz Bruker Avance NEO spectrometers equipped with a cryogenic TCI probe and a 600 MHz Bruker Avance spectrometer, at 25 °C in H_2_O/D_2_O (90:10, v/v). Concentrations of the solutions were 20 mM. All parameters of NMR measurements are presented in Table S4. Two-dimensional NMR spectra were processed with TOPSPIN (Bruker) and analyzed with SPARKY (Goddard and Kneller 2000) programs. Complete assignments of the ^1^H, ^15^N, and ^13^C resonances for all the peptides were done by application of a standard procedure (Wüthrich 1986) based on inspection of the 2D experiments: ^1^H-^1^H TOCSY (Braunschweiler and Ernst 1983) (with mixing times 80 and 90 ms), ^1^H-^1^H ROESY (Bax and Davis 1985) (with mixing time 100 and 300 ms), ^1^H-^13^C HSQC (Bodenhausen and Ruben 1980) focused on the aromatic and aliphatic regions separately, and ^1^H-^15^N HSQC (Bodenhausen and Ruben 1980). Through space distances between protons were determined by analysis of the 2D ^1^H-^1^H ROESY spectra inter proton cross peaks. The lowest-energy structures of the peptides studied were calculated with the XPlor package (Schwieters et al. 2003). It is a widely used software package used for biomolecular structure determination from NMR and other data sources.

### Radioligand receptor binding assays

The binding affinity of cyclic and linear enkephalin analogs for μ-opioid receptor (MOR) was determined in competitive radioligand binding assays using membrane preparations from rat brain homogenates. The homogenates were obtained as described previously (Matalińska et al. 2020). The membrane preparations were incubated at 25 °C for 60 min in the presence of 1.0 nM [^3^H]DAMGO (obtained from PerkinElmer, USA) and the set of concentrations of the assayed compound proper for a particular compound and type of determination (screening or a full-displacement curve).

For screening purposes, we performed the experiments with the following concentrations of the compounds tested: 30 μM, 10 μM, and 3 μM. For full-displacement curves, 10 concentrations ranging either from 0.03 nM to 1 μM or from 300 nM to 30 μM were used. The range was chosen for the single compounds in a manner such that the expected IC_50_ would fall in the middle of the range. In the case of several experiments, only five concentrations were used (they are marked in the results table).

Non-specific binding was measured in the presence of 10 μM naloxone. The assays were conducted with the assay buffer made of 50 mM Tris–HCl (pH 7.4), bacitracin (100 μg/ml), bestatin (30 μM), and captopril (10 μM), phenylmethylsulfonyl fluoride (PMSF, 30 μg/ml) in a total volume of 0.5 ml. After the incubation, a rapid filtration with a M-24 Cell Harvester (Brandel/USA) through GF/B Whatman glass fiber strips was conducted. The filters were soaked with 0.5% PEI just before the harvesting so as to minimize the extent of non-specific binding. Filter disks were cut from the sheet and placed separately in 24-well plates. The Optiphase Supermix scintillation solution (Perkin Elmer, USA) was added to each well. Radioactivity was measured in a scintillation counter MicroBeta LS, Trilux (PerkinElmer, USA). Displacement curves were drawn and the mean IC_50_ values were determined with SEMs (GraphPad Prism v. 5.0, San Diego, CA).

The results (of full concentration range measurements) are means ± standard error of the mean of two or three independent experiments done with two repetitions, if not stated otherwise. The results of screening experiments are means ± standard deviation of two independent experiments done with three repetitions, if not stated otherwise.

### Quantum chemical calculations

The structure of c-(Tyr-*m*-Phe-*p*)-M-NH_2_ as calculated by XPLOR was subject to quantum mechanical structure optimization. The calculations were done using Gaussian 09 (Frisch et al. 2013). The initial geometry was minimized at the B3LYP/6-31G(d,p) level in the gas phase. The obtained structure which exhibited proton transfer between NH_3_^+^ group of Tyr^1^ and the adjacent C = O group was modified by moving the proton back to the amino group and subjected to optimization at the B3LYP/6-31G(d,p) level with the PCM solvent model (Mennucci 2018). The obtained geometry served harmonic frequency calculation at the same level to check if it is a minimum (no imaginary frequencies). Atomic coordinates of the starting point geometry and the QM optimized structure are given in Supplementary Materials (Listings S1 and S2).

### Molecular docking

Molecular docking was performed in AutoDock Vina (Trott and Olson 2010). The structure of the linear peptide [Met^5^]enk-NH_2_ (in extended conformation) was prepared in Biovia Discovery Studio Visualizer (Dassault Systèmes 2018). The structure of the biaryl c-(Tyr-*m*-Phe-*p*)-M-NH_2_ was the one resulting from the quantum chemical optimization started from the NMR-derived geometry, as described in the previous section. The MOR structure used for docking was 8EFQ structure (Zhuang et al. 2022). The protein preparation was done in Biovia Discovery Studio Visualizer by removing the experimental ligand and G-protein, as well as by adding hydrogens. The protonation states were set as expected at pH ~ 7. The docking box was set to encompass the binding site of MOR but significantly extended (box sizes: 41.0 Å × 32.7 Å × 33.7 Å). The receptor structure was treated as rigid. In the case of [Met^5^]enk-NH_2_, full ligand flexibility (except for amide bonds) was allowed. In the case of the biaryl **c-(Tyr-*m*-Phe-*p*)-M-NH**_**2**_, the exocyclic bonds (except for amide bonds) were treated as flexible. The docking exhaustiveness was set to 20. The top docking results were inspected visually. The docking scores are given in Tables S4 and S5 in Supplementary Information. In the case of [Met^5^]enk-NH_2_, a pharmacophoric filter was applied according to which poses without Asp3.32∙∙∙Tyr^1^ NH_3_^+^ interaction were discarded from analysis. Such interaction is expected for typical high affinity peptide opioids based on experimental data from mutagenetic studies (Li et al. 1999; Chavkin et al. 2001). Molecular graphics were prepared in Biovia Discovery Studio Visualizer and in open-source PyMol (Version 2.0 Schrödinger, LLC).

## Results and discussion

### Chemistry

**Scheme 1.**
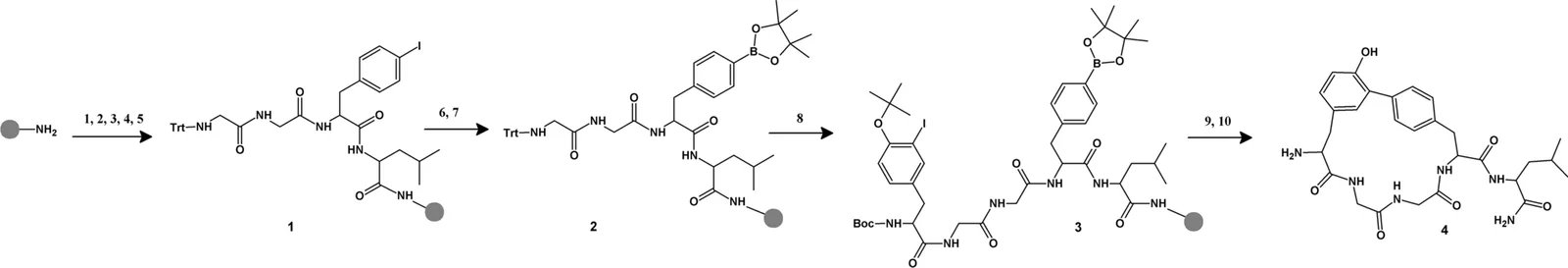
Synthesis of biaryl bridged cyclic enkephalin analog with the phenylalanine derivative at position 4 by the example of H-(cyclo-*m,p*)-[Tyr-Gly-Gly-Phe]-Leu-NH_2_. Reaction conditions: (1) Fmoc-amino acid, HATU in the presence of HOBt and DIEA in DMF, rt; (2) piperidine/DMF (1:3), 2 × 10 min; (3) repeated deprotection and amino acid coupling; (4) piperidine/DMF (1:3), 2 × 10 min; (5) TrCl, DIEA, DMF, rt, 3 h (**1**); (6) PdCl_2_(dppf)_2_∙CH_2_Cl_2_, dppf, B_2_pin_2_, KOAc, DMSO, 80 °C, 4–8 h; (7) TFA/H_2_O/CH_2_Cl_2_ (0.2:1:98.8), 20 min (**2**); (8) Boc-Tyr(.^*t*^Bu,3-I)-OH, HATU in the presence of HOBt and DIEA in DMF, rt (**3**); (9) PdCl_2_(dppf)_2_∙CH_2_Cl_2_, dppf, CsF, dioxane/water, 80 °C. (10) TFA/TIS/H_2_O, rt, 3 h (**4**)

Synthesis of cyclic enkephalin analogs bearing biaryl bond was based on solid-phase Miyaura–Suzuki macrocyclization of linear peptides containing both the boronate and iodinated analogs of aromatic amino acids—phenylalanine and tyrosine. From a synthetic point of view, the obtained compounds can be divided into two groups—either with analogs of phenylalanine or tyrosine at a fourth position of the peptide chain. In the case of the first group (Scheme 1), the synthesis was based on obtaining the C-terminal tetrapeptide fragment of the target compound, with iodinated Phe, protected at the N-terminus with a trityl group. The obtained tetrapeptide fragment was subjected to Miyaura reaction on a solid support as described in (Afonso et al. 2010, 2012). The Miyaura borylation reaction was carried out in DMSO at 80 °C for 4–8 h using bispinacolatodiboron (B_2_pin_2_, 4 eq), PdCl_2_(dppf)_2_∙CH_2_Cl_2_ (0.18 eq), dppf (0.09 eq), and KOAc (6 eq) as a base. After carrying out a borylation reaction and removing the trityl group, the elongation of the peptide chain was continued until attaching a terminal iodine derivative of phenylalanine or tyrosine, protected at the N-terminus with a Boc or Fmoc group. In the case of peptides with the N-terminal phenylalanine derivative, the Fmoc group was cleaved and trityl protection was introduced.

**Scheme 2.**
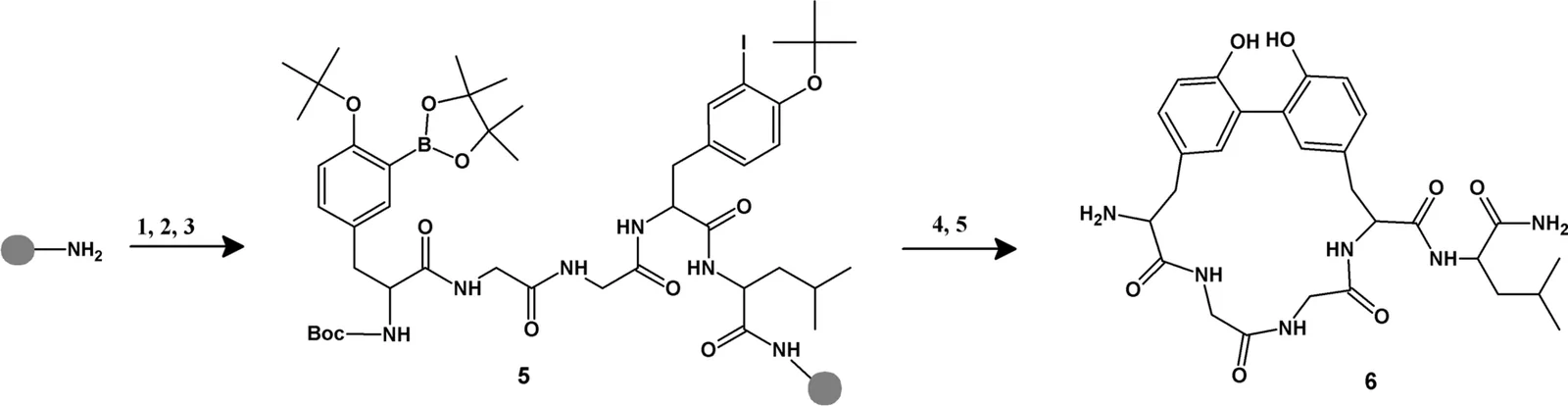
Synthesis of biaryl bridged cyclic enkephalin analogs with the tyrosine derivative at position 4 by the example of H-(cyclo-*m,m*)-[Tyr-Gly-Gly-Tyr]-Leu-NH_2_. Reaction conditions: (1) Fmoc-amino acid, HATU in the presence of HOBt and DIEA in DMF, rt; (2) piperidine/DMF (1:3), 2 × 10 min; (3) repeated deprotection and amino acid coupling (**5**); (4) Pd(dppf)Cl_2_∙CH_2_Cl_2_, dppf, CsF, dioxane/water, 80 °C; (5) TFA/TIS/H_2_O, rt, 3 h (**6**)

As regards the peptides with the Tyr residue at a fourth position (Scheme 2), the Miyaura reaction on the resin with N-trityl protected, *t*-butyl ether of iodinated tyrosine gave a low yield. Therefore, the boronic derivative was introduced at the end of the amino acid sequence in the form of one of two synthesized amino acid residues: Boc-*L*-Tyr(^*t*^Bu,3-I)-OH and Boc-*L*-Phe(4-Bpin)-OH (Malan and Morin 1998). Peptides containing both the boronate and iodinated analogs of aromatic amino acids were cyclized at 80 °C for 18–24 h by solid supported Suzuki coupling using Pd(dppf)Cl_2_∙CH_2_Cl_2_, dppf, base, and degassed dioxane/water (9:1) as a solvent.

### NMR studies

Important conformational information on the peptides studied can be obtained from the [^1^H-^1^H] ROESY spectra. Apart of their contribution to the assignment of the ^1^H, ^15^N, and ^13^C resonances, they were used in the process of determining the peptides spatial structures because they allowed us to detect the nontrivial, inter-residue NOE contacts. Such numerous contacts were found for all the peptides investigated. They are presented in Table S1 in Supplementary Information. It was assumed that if there is a contact between two protons in the spectrum, they are at a distance not greater than 5.5 Å.

**Fig. 2:**
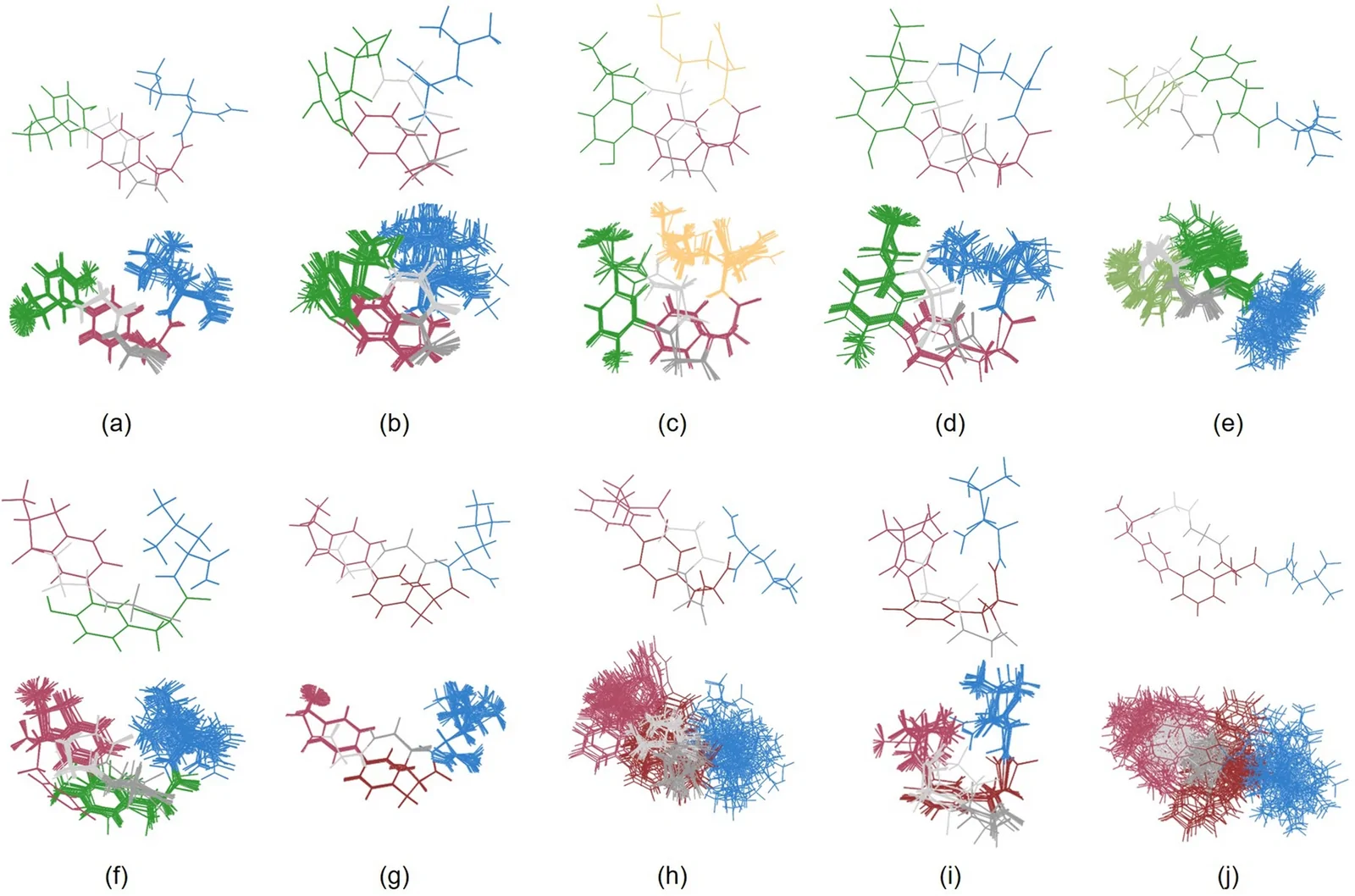
Lowest-energy structures and ensembles of the 50 lowest-energy ones of: (**a**) c-(Tyr-m-Phe-p)-L-NH2; (**b**) c-(Tyr-m-Phe-m)-L-NH2 (**c**) c-(Tyr-m-Phe-p)-M-NH2; (**d**) c-(Tyr-m-Phe-p)-L-OH; (**e**) c-(Tyr-m-Tyr-m)-L-NH2; (**f**) c-(Phe-p-Tyr-m)-L-NH2; (**g**) c-(Phe-p-Phe-p)-L-NH2; (**h**) c-(Phe-m-Phe-p)-L-NH2; (**i**) c-(Phe-m-Phe-m)-L-NH2; (**j**) c-(Phe-p-Phe-m)-L-NH2. Glycine—gray (the first Gly residue in the sequence is brighter and the next one is darker), leucine—blue, methionine—yellow, phenylalanine—red, tyrosine—green

The NOE contacts were used as the input data for calculations of the lowest-energy conformations. Using the XPLOR program (Schwieter et al. 2003), five hundred structures were generated for each of the peptide studied, from which sets of fifty lowest-energy ones were then selected. The structures in each set were superimposed in PyMOL and the conformation with the lowest energy was separated. RMSDs (root-mean-square deviations) and torsion angles were calculated with MOLMOL (Koradi et al. 1996). The results of these calculations are shown in Table 2 and Fig. 2.

**Table 2:** Torsion angles values of the peptides studied calculated on the basis of NMR parameters with the XPlor program and RMSDs (root-mean-square-deviations)

Peptide	Residue										RMSD^a^ [Å]
	1		2		3		4		5		
	φ	ψ	φ	ψ	φ	ψ	φ	ψ	φ	ψ	
c-(Tyr-*m*-Phe-*p*)-L-NH_2_	–	135.0° (± 10.0°)	126.4° (± 11.9°)	24.7° (± 4.1°)	−89.7° (± 5.5°)	−16.8° (± 14.2°)	−94.4° (± 20.1°)	3.8° (± 5.3°)	−48.9° (± 8.5°)	–	0.073
c-(Tyr-*m*-Phe-*m*)-L-NH_2_	–	−101.5° (± 33.8°)	176.7° (± 42.5°)	−11.2° (± 5.6°)	−100.6° (± 7.4°)	−84.5° (± 5.0°)	−67.0° (± 3.6°)	−35.4° (± 32.6°)	−25.1° (± 59.9°)	–	0.064
c-(Tyr-*m*-Phe-*p*)-M-NH_2_	–	−125.8° (± 44.2°)	94.6° (± 31.7°)	−20.3° (± 65.8°)	−13.1° (± 55.0°)	−11.8° (± 3.0°)	−157.0° (± 11.9°)	−62.6° (± 4.2°)	−43.5° (± 22.3°)	–	0.202
c-(Tyr-*m*-Phe-*p*)-L-OH	–	−31.7° (± 8.2°)	56.9° (± 5.9°)	62.7° (± 10.5°)	176.3° (± 26.2°)	148.3° (± 11.1°)	32.0° (± 2.5°)	−70.2° (± 49.9°)	−71.3° (± 51.5°)	–	0.068
c-(Tyr-*m*-Tyr-*m*)-L-NH_2_	–	−56.3° (± 56.4°)	91.5° (± 66.3°)	5.1° (± 6.3°)	−99.1° (± 14.3°)	16.1° (± 40.0°)	−40.5° (± 39.5°)	137.3° (± 47.7°)	−65.2° (± 68.9°)	–	0.115
c-(Phe-*p*-Tyr-*m*)-L-NH_2_	–	−53.2° (± 35.8°)	87.3° (± 87.8°)	−34.0° (± 75.3°)	−101.0° (± 51.9°)	−33.6° (± 43.1°)	−57.3° (± 21.9°)	−30.7° (± 17.6°)	−71.3° (± 64.7°)	–	0.398
c-(Phe-*p*-Phe-*p*)-L-NH_2_	–	−25.9° (± 0.7°)	−164.0° (± 1.5°)	−46.3° (± 2.0°)	78.9° (± 1.4°)	−15.2° (± 3.6°)	−48.5° (± 4.3°)	−16.5° (± 0.8°)	36.9° (± 2.2°)	–	0.015
c-(Phe-*m*-Phe-*p*)-L-NH_2_	–	133.5° (± 8.0°)	−173.4° (± 50.7°)	51.7° (± 70.6°)	−63.9° (± 37.3°)	40.1° (± 57.4°)	−148.3° (± 36.7°)	100.2° (± 67.5°)	−27.1° (± 71.6°)	–	0.532
c-(Phe-*p*-Phe-*m*)-L-NH_2_	–	−139.7° (± 85.9°)	168.9° (± 96.3°)	27.9° (± 86.3°)	−168.3° (± 73.6°)	129.9° (± 79.3°)	−156.6° (± 99.0°)	57.4° (± 68.6°)	−32.9° (± 70.4°)	–	0.571
c-(Phe-*m*-Phe-*m*)-L-NH_2_	–	−16.7° (± 76.4°)	−131.0° (± 67.2°)	−66.5° (± 39.9°)	−143.5° (± 53.2°)	20.7° (± 13.4°)	−130.0° (± 13.3°)	−17.9° (± 40.6°)	−155.9° (± 57.8°)	–	0.323

^a^RMSD of atomic positions of the ensemble, calculated using backbone atoms of residues 1–4

Except peptides **c-(Phe-*m*-Phe-*p*)-L-NH**_**2**_ and **c-(Phe-*p*-Phe-*m*)-L-NH**_**2**_, the obtained conformations are well defined as confirmed by very low RMSD values (Table 2). It can therefore be concluded that for these analogs there is a preferred conformation in which the molecule reaches its energy minimum. Residues 1–4 are more rigid due to cyclization while the fifth amino acid residue has more conformational freedom than the rest of the molecule. However, for example in **c-(Tyr-*m*-Phe-*p*)-M-NH**_**2**_, a partial rigidification of atoms of the C-terminal methionine residue can be observed (Fig. 2c).

**Fig. 3:**
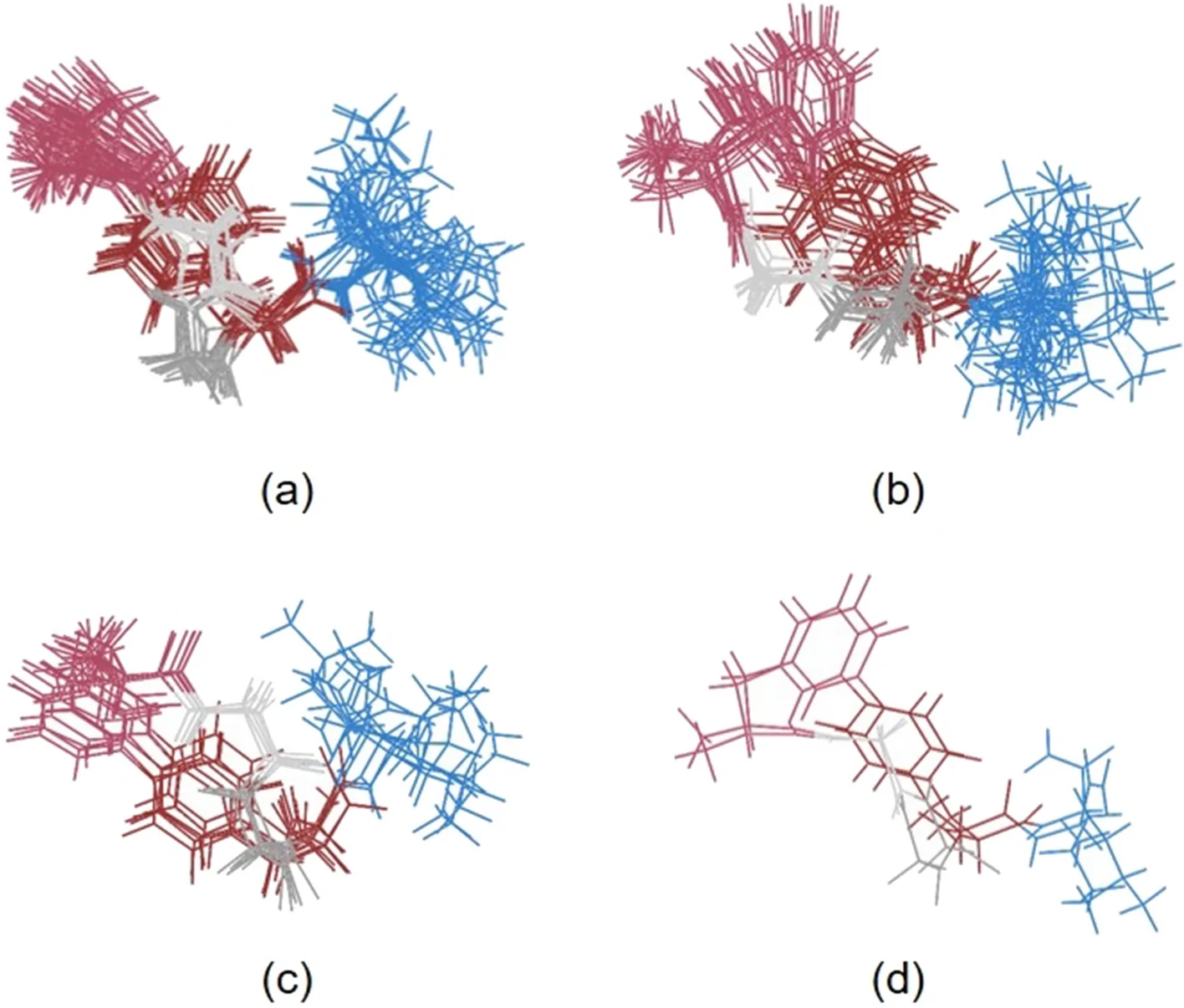
Extracted four groups of conformers of c-(Phe-*m*-Phe-*p*)-L-NH_2_ arranged according to increasing energy

In the case of **c-(Phe-*m*-Phe-*p*)-L-NH**_**2**_, a closer inspection of the 50 lowest-energy structures allowed us to distinguish four conformational families of that peptide. It was done by superimposition of the structures based on their cyclic fragments and looking for differences between them. The group of structures with the lowest energies is the most populated and the number of structures in other groups decreases with increasing energy. The isolated conformational families of **c-(Phe-*m*-Phe-*p*)-L-NH**_**2**_ are shown in Fig. 3. For **c-(Phe-*p*-Phe-*m*)-L-NH**_**2**_, it was not possible to create groups containing more than two structures which proves that it has a greater conformational freedom than other analogs.

Closing of the enkephalin peptide chain by formation of the biaryl moiety forces, it into a folded conformation of the β-turn type. In the case of enkephalins, there are two β-turns possible, with either residues 2 and 3 or residues 3 and 4 sitting at the *i* + *1* and *i* + *2* positions of a β-turn, respectively. It was found from the calculations that generally no intramolecular hydrogen bonds are present in the peptides studied. The Met^5^ NH∙∙∙Gly^2^ C = O hydrogen bond was found in 20 from 50 lowest-energy structures of **c-(Tyr-*m*-Phe-*p*)-M-NH**_**2**_, but these are mainly higher-energy conformations and a few of lower energy, so it is difficult to say that a hydrogen bond is distinctly present in that analog. A similar situation takes place for **c-(Phe-*p*-Tyr-*m*)-L-NH**_**2**_, but here a hydrogen bond (Leu^5^ NH∙∙∙Gly^2^ C = O) is present in 19 highest-energy structures from the group of 50. The only peptide worth attention in that respect is **c-(Phe-*m*-Phe-*m*)-L-NH**_**2**_ where two hydrogen bonds—Leu^5^ NH∙∙∙Gly^2^ C = O and Leu^5^ CO-NH_2_∙∙∙Phe^1^ C = O—are present in 8 lowest-energy structures from the 50 analyzed. The values of the dihedral angles presented in Table 1 show that in all the peptides studied, only β-turn of type IV (in which there is no hydrogen bond), according to the classification of Lewis et al. (1973), is possible at positions 2 and 3, except **c-(Tyr-*m*-Tyr-*m*)-L-NH**_**2**_, where type I’ β-turn may be present. As regards positions 3 and 4, **c-(Tyr-*m*-Phe-*p*)-L-NH**_**2**_, **c-(Phe-*p*-Tyr-*m*)-L-NH**_**2**_, and **c-(Phe-*p*-Phe-*p*)-L-NH**_**2**_ can adopt the type III β-turn, whereas only type IV is possible in all other peptides. The dihedral angles obtained for **c-(Phe-*m*-Phe-*m*)-L-NH**_**2**_ suggest the presence of only type IV β-turn, regardless of 2–3 or 3–4 location what is in contradiction with the presence of the Leu^5^ NH∙∙∙Gly^2^ C = O hydrogen bond found in 8 lowest-energy structures of that peptide. The presence of type IV β-turn in the peptides studied is in agreement with a general absence of intramolecular hydrogen bonds.

As regards the multiplicities of NMR signals of the peptides studied, most of them corresponds very well with the theory. However, there are some unusual patterns (indicated by question marks in the assignments of the NMR signals in the spectra of cyclic peptides in Supplementary Information). They are very often a result of the signals overlap which made it very difficult to find unequivocally the kind of a multiplet. In the case of NH protons of glycine residues, it happened sometimes that broad, low singlets (described as “broad s”) appeared, instead of triplets. Moreover, in most of the peptides (**c-(Tyr-*m*-Tyr-*m*)-L-NH**_**2**_, **c-(Tyr-*m*-Phe-*m*)-L-NH**_**2**_, **c-(Tyr-*m*-Phe-*p*)-L-NH**_**2**_, **c-(Phe-*p*-Tyr-*m*)-L-NH**_**2**_, **c-(Phe-*m*-Phe-*m*)-L-NH**_**2**_, and **c-(Phe-*p*-Phe-*p*)-L-NH**_**2**_), additional couplings of aromatic protons were detected, which do not result directly from the compound structure. In **c-(Phe-*m*-Phe-*m*)-L-NH**_**2**_ in turn, between theoretically different but in fact very similar protons gave a triplet in the place of a predicted doublet of doublets. Unusual multiplets were also observed for α protons of Gly residues in **c-(Phe-*p*-Phe-*m*)-L-NH**_**2**_. There are doublets where theoretically doublets of doublets should be present. It can be related with the broadening of NH proton signals of both Gly residues in this peptide. This broadening may result from the presence of many conformations in the peptide’s conformational equilibrium. It is in agreement with a very large number of various calculated structures of **c-(Phe-*p*-Phe-*m*)-L-NH**_**2**_ which indicates its large conformational freedom. The Gly–Gly dipeptide fragment may play here a substantial role.

**Fig. 4:**
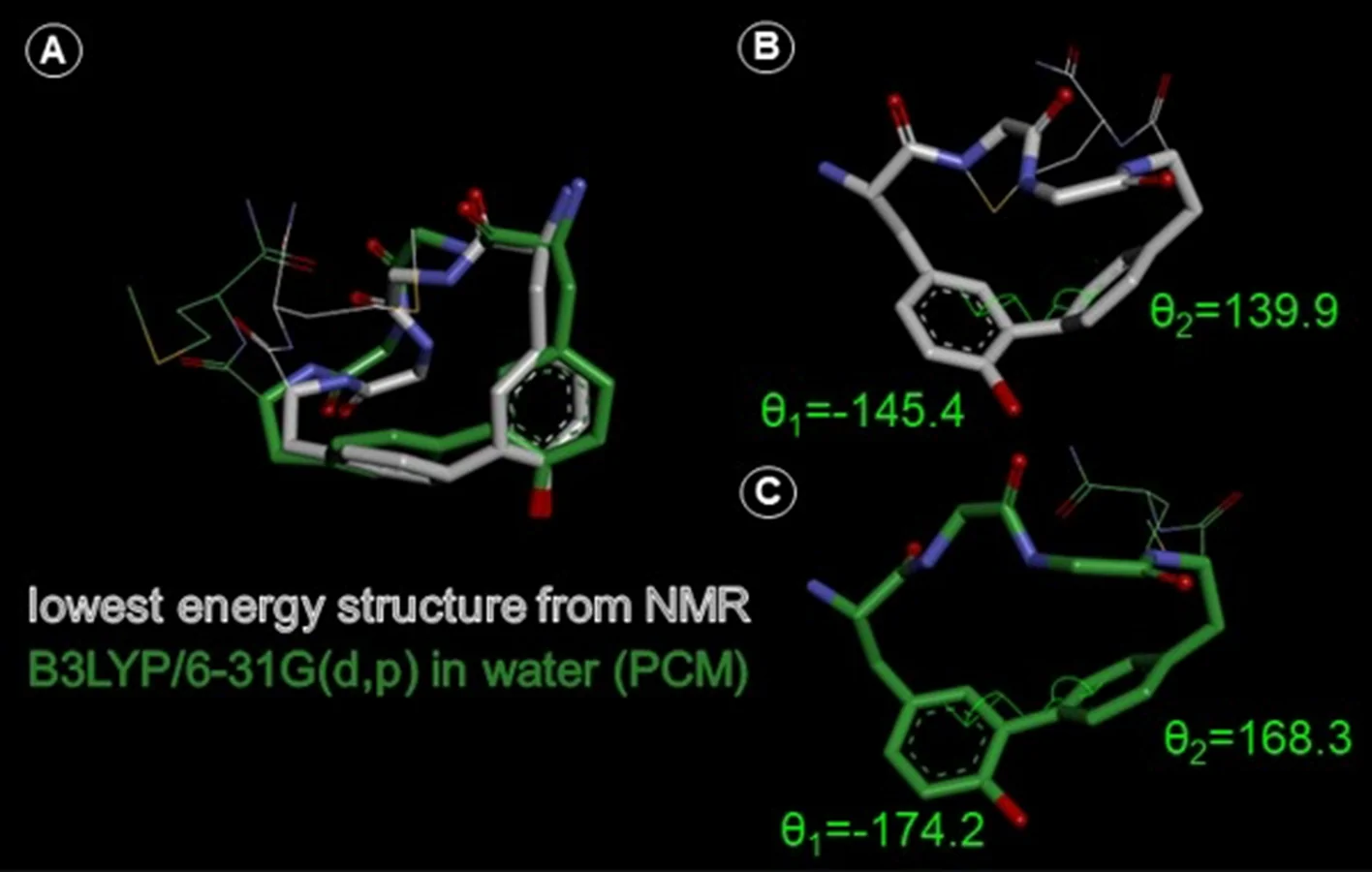
Comparison of c-(Tyr-*m*-Phe-*p*)-M-NH_2_ structures obtained by XPLOR calculations (white) and upon additional QM minimization (green). (**a**) Superposition of both structures, (**b**) XPLOR lowest-energy structure, (**c**) QM minimized structure. In (**b**) and (**c**) the marked dihedral angles *θ*_1_ and *θ*_2_ and their values are shown to illustrate the change in the pyramidalization of aromatic carbons upon QM minimization. Met^5^ side chain and the C-terminal amide fragment are shown as thin lines for clarity. Hydrogen display is suppressed

Close inspection of the XPLOR-calculated three-dimensional structures of the studied peptides (see 3-D_structures.zip file in Supplementary Information) shows a significant distortion in the geometry around the biaryl linkage. The aromatic carbons joining the rings are pyramidalized to some extent (an example in Fig. 4b). This is observed in all the peptides except for **c-(Phe-*p*-Phe-*p*)-L-NH**_**2**_ and **c-(Phe-*p*-Phe-*m*)-L-NH**_**2**_. Similar examples of pyramidalized aromatic carbons in cyclic molecules with biaryl fragment are known in literature (Cochrane et al. 2012; Zhao et al. 2018; Zhu et al. 2018). On the other hand, we suspected that XPLOR force field may not be entirely suitable for modeling the biaryl fragment. We were then curious to see if this pyramidalization persists after quantum mechanical (QM) geometry optimization. To explore this possibility, we subjected the XPLOR-obtained lowest-energy structure of **c-(Tyr-*m*-Phe-*p*)-M-NH**_**2**_ to QM optimization at the B3LYP/6-31G(d,p) level in water (PCM implicit solvent model (Mennucci 2012)). As a result, the distortion in the biaryl junction was significantly but not entirely relieved (Fig. 4c). At the same time, the rest of the molecule retains its overall shape and conformation (superposition of the structures Fig. 4a, comparison of the dihedral angles Table S2). This suggests that the biaryl fragment may indeed have some distorted geometry although XPLOR modeling could overestimate it due to lack of proper parametrization. This issue warrants further investigation beyond the scope of this report.

One of the features of substituted biaryl compounds is their ability to appear as atropisomers. In the case of peptides discussed in this paper, such an isomerism can occur only in compounds with the biaryl bridge of the *para*-*para* configuration. The only peptide with such a configuration of the biaryl bridge is **c-(Phe-*p*-Phe-*p*)-Leu-NH**_**2**_. The aromatic rings of phenylalanine residues in that peptide can undergo a rotation around the bond joining them. If this rotation was hindered, then chemical shifts of both δ and ε protons in the aromatic rings of the Phe residues should be different. Instead, δ and ε protons in both aromatic rings of **c-(Phe-*p*-Phe-*p*)-Leu-NH**_**2**_ give single, averaged signals which shows a free rotation of the rings. It is consistent with the lack of aromatic carbon atoms pyramidalization in this peptide. Otherwise distorted bond geometry of γ and/or ζ aromatic carbons would severely limit a free aromatic rings rotation.

### CD studies

Analysis of CD spectra of enkephalins is difficult due to the presence of two aromatic amino acid residues. Such residues give large CD bands in the far-UV region originating from their ^1^L_a_ transitions. They are usually positive and do not depend strongly on the peptide conformation (Woody 1978). These bands overlap the signals of the peptide chromophores which give information on the secondary structure of peptides and proteins hindering the conformational analysis. The situation is even more complicated in the case of biaryl peptides like the ones discussed in this paper. A direct connection of two aromatic rings with each other can result in exciton coupling of their electronic transitions. The exciton coupling is a very useful tool of configurational and conformational studies (Harada and Nakanishi 1972; Pescitelli 2022), but when aromatic exciton bands appear in the region of peptide chromophores absorption, they additionally obscure the far-UV region and make CD analysis of peptides more difficult.

**Fig. 5:**
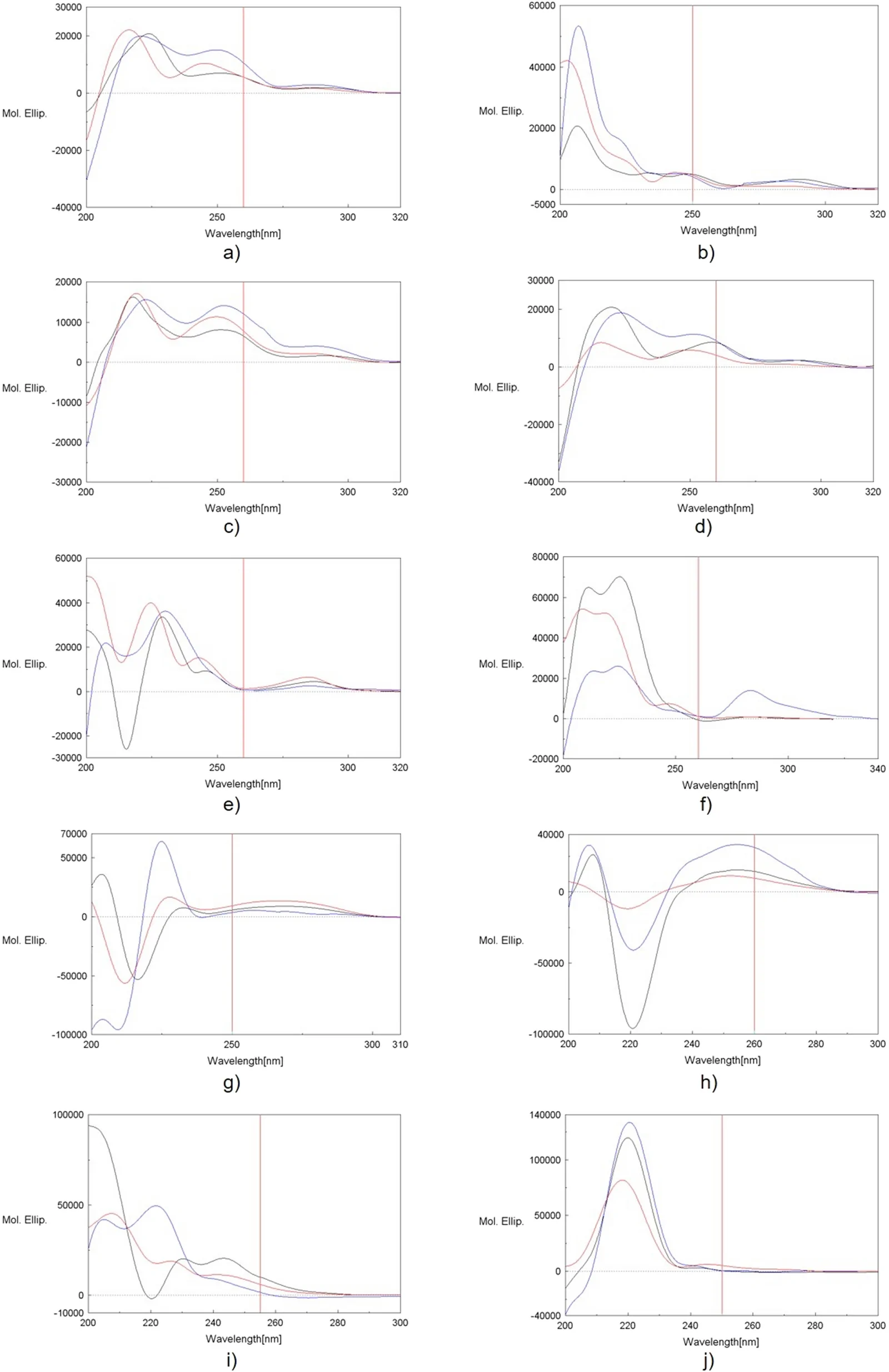
CD spectra of biaryl enkephalin analogs in MeOH (black), TFE (red), and water, pH 7 (blue): (**a**) c-(Tyr-*m*-Phe-*p*)-L-NH_2_; (**b**) c-(Tyr-*m*-Phe-*m*)-L-NH_2_; (**c**) c-(Tyr-*m*-Phe-*p*)-M-NH_2_; (**d**) c-(Tyr-*m*-Phe-*p*)-L-OH; (**e**) c-(Tyr-*m*-Tyr-*m*)-L-NH_2_; (**f**) c-(Phe-*p*-Tyr-*m*)-L-NH_2_; (**g**) c-(Phe-*p*-Phe-*p*)-L-NH_2_; (**h**) c-(Phe-*m*-Phe-*p*)-L-NH_2_; (**i**) c-(Phe-*p*-Phe-*m*)-L-NH_2_; (**j**) c-(Phe-*m*-Phe-*m*)-L-NH_2_

In light of the above, the CD spectra of the biaryl enkephalin analogs (Fig. 5) do not allow to draw reliable conformational conclusions. However, in this case, they can show conformational differences between individual analogs and provide information on their rigidity on the basis of their solvent-dependence. But when comparing conformations of the peptides studied a proper caution should be observed because the differences between their CD spectra may result not so much from their conformational differences as from varying aromatic contributions. There is no such problem in the case of **c-(Tyr-*m*-Phe-*p*)-L-NH**_**2,**_** c-(Tyr-*m*-Phe-*p*)-M-NH**_**2**_, and **c-(Tyr-*m*-Phe-*p*)-L-OH**. These analogs have the same biaryl ring structure and differ only by their last amino acid residue and the C-terminal group. The CD spectra of those peptides (Fig. 5a, c, d) show that their conformations are similar and they are not influenced significantly by these structural differences. Instead, a change of the biaryl bridge configuration from *meta*–*para* to *meta*–*meta* leads to a change of the CD spectrum resulting from decreasing of the biaryl ring size and hence its different conformation. It can be seen by comparison of the spectra of **c-(Tyr-*m*-Phe-*p*)-L-NH**_**2**_ (Fig. 5a) and **c-(Tyr-*m*-Phe-*m*)-L-NH**_**2**_ (Fig. 5b). Effect of different biaryl bridge configurations and ring sizes is also distinctly reflected in the spectra of **c-(Phe-*p*-Phe-*p*)-L-NH**_**2**_ (Fig. 5g), **c-(Phe-*m*-Phe-*p*)-L-NH**_**2**_ (Fig. 5h), **c-(Phe-*p*-Phe-*m*)-L-NH**_**2**_ (Fig. 5i), and **c-(Phe-*m*-Phe-*m*)-L-NH**_**2**_ (Fig. 5j).

The smallest solvent-dependence of CD spectra is shown by **c-(Phe-*p*-Phe-*m*)-L-NH**_**2**_ (Fig. 5i). It shows that the conformation of that peptide is quite stable. This is somewhat surprising because that very peptide showed the greatest conformational variability during XPlor calculations on the basis of NMR parameters. Smaller, comparable conformational stability can be observed for **c-(Tyr-*m*-Phe-*p*)-L-NH**_**2**_ (Fig. 5a), **c-(Tyr-*m*-Phe-*m*)-L-NH**_**2**_ (Fig. 5b), **c-(Tyr-*m*-Phe-*p*)-M-NH**_**2**_ (Fig. 5c), and **c-(Tyr-*m*-Phe-*p*)-L-OH** (Fig. 5d). Other analogs seem to exhibit a larger conformational freedom.

### μ-Opioid receptor affinity

Seven of the synthesized biaryl enkephalin analogs and their linear counterparts were tested for MOR affinity in radioligand displacement assays. The results are shown in Table 3 and Table S3.

**Table 3:** Affinity of the studied analogs for the μ-opioid receptor

Peptide	IC_50_ ± S.E.M. [nM]^a^	*n* exp^b^	Radioligand SB at 30 μM of ligand^c^
c-(Tyr-*m*-Phe-*p*)-L-NH_2_	> 30 000^d^	2	77 ± 4
c-(Tyr-*m*-Phe-*p*)-M-NH_2_	1372.5 ± 266.5	3	12 ± 4
c-(Phe-*p*-Phe-*p*)-L-NH_2_	> 30 000^d^	2	73 ± 19
c-(Tyr-*m*-Tyr-*m*)-L-NH_2_	> > 30 000^d^	2	117 ± 1
c-(Phe-*p*-Tyr-*m*)-L-NH_2_	6671.0 ± 262.0	2^e^ + 1	17 ± 3
c-(Tyr-*m*-Phe-*p*)-L-OH	10 000 < IC_50_ < 30 000^d^	2	36 ± 6
c-(Tyr-*m*-Phe-*m*)-L-NH_2_	3085.0^f^	1 + 2	12 ± 3
[Leu^5^]enk-OH	17.8 ± 3.8	3	n/d^g^
[Leu^5^]enk-NH_2_	6.1 ± 1.4	3	n/d^g^
[Tyr^4^,Leu^5^]enk-NH_2_	221.7 ± 15.9	2	n/d^g^
[Phe^1^,Tyr^4^,Leu^5^]enk-NH_2_	10 000 < IC_50_ < 30 000^d^	2 + 2	43 ± 11
[Phe^1^,Leu^5^]enk-NH_2_	595.8 ± 48.8	2 + 1	5 ± 1
[Met^5^]enk-NH_2_	2.6 ± 0.2	3	n/d^g^

^a^Half-maximal inhibitory concentration with standard error of the mean; if not stated otherwise, it is a mean of 2 or 3 independent experiments done with two replicates

^b^Number of experiments; if more than one number is given, the first stands for number of experiments with full range of concentrations and the second stands for number of screening experiments

^c^% of radioligand specific binding at 30 μM of ligand; mean and standard deviation of two determinations done with three replicates; data for all screening concentrations (30 μM, 10 μM and 3 μM) are given in Table S3

^d^Estimation based on the screening results with the following concentrations 30 μM, 10 μM, and 3 μM (see Table S3 for numerical data)

^e^Based on shorter (5 points) range of concentrations

^f^Based on one experiment (done with two replicates), with shorter (5) range of concentrations

^g^Not determined

All the tested biaryl analogs exhibit low or very low MOR affinity with IC_50_ in the micromolar range. The best binding is shown by **c-(Tyr-*m*-Phe-*p*)-M-NH**_**2**_ (IC_50_ = 1372.5 nM), and it is however cca 500-times weaker than that of its linear counterpart **[Met**^**5**^**]enk-NH**_**2**_ (IC_50_ = 2.6 nM). The analog **c-(Tyr-*m*-Phe-*p*)-L-NH**_**2**_, which is different from the former only due to Met/Leu exchange in the fifth position, is a much weaker MOR ligand (IC_50_ > 30 000 nM). If the mode of cyclization is changed (**c-(Tyr-*m*-Phe-*m*)-L-NH**_**2**_), the affinity improves (IC_50_ = 3085.0 nM), but again, this analog is cca 500 (weaker than the linear counterpart (**[Leu**^**5**^**]enk-NH**_**2**_, IC50 = 6.1 nM). The acid **c-(Tyr-*m*-Phe-*p*)-L-OH** (10 000 < IC50 < 30 000 is not much better than the amide **c-(Tyr-*m*-Phe-*p*)-L-NH**_**2**_ and it is drastically worse than the linear acid **[Leu**^**5**^**]enk-OH** (IC50 = 17.8 nM). Placing Tyr in position four (**c-(Tyr-*m*-Tyr-*m*)-L-NH**_**2**_) abolishes any MOR binding. This modification is also not favorable in linear analogs, as **[Tyr**^**4**^**,Leu**^**5**^**]enk-NH**_**2**_ exhibits binding reduced by about 40 times (IC50 = 221.7 nM) compared to the parent **[Leu**^**5**^**]enk-NH**_**2**_.

Interestingly, the biaryl analog devoid of Tyr in the first position, **c-(Phe-*p*-Tyr-*m*)-L-NH**_**2**_ exhibits binding slightly better (IC_50_ = 6671.0 nM) than the linear **[Phe**^**1**^**,Tyr**^**4**^**,Leu**^**5**^**]enk-NH**_**2**_ (10 000 < IC_50_ < 30 000). This is (remotely) consistent with the fact that while Tyr in the first position is usually perceived as critical to high affinity MOR binding, there were a few previous reports for cyclic analogs in which exocyclic Phe in the first position did not negatively affect affinity yielding nanomolar analogs (Weltrowska et al. 2008; Burden et al. 1999).

### Molecular docking

In the desire to understand low MOR affinity of the biaryl analogs, we modeled the linear **[Met**^**5**^**]enk-NH**_**2**_ and its cyclic counterpart **c-(Tyr-*m*-Phe-*p*)-M-NH**_**2**_ in the binding site of the receptor. Both peptides were docked to 8EFQ structure of MOR (Zhuang et al. 2022). This structure seems particularly suitable for modeling of enkephalins in MOR, as the experimental ligand present therein is an enkephalin-related, MOR selective agonist **DAMGO** ([d-Ala^2^,*N*-MePhe^4^,Gly^5^-ol]-enkephalin).

**Fig. 6:**
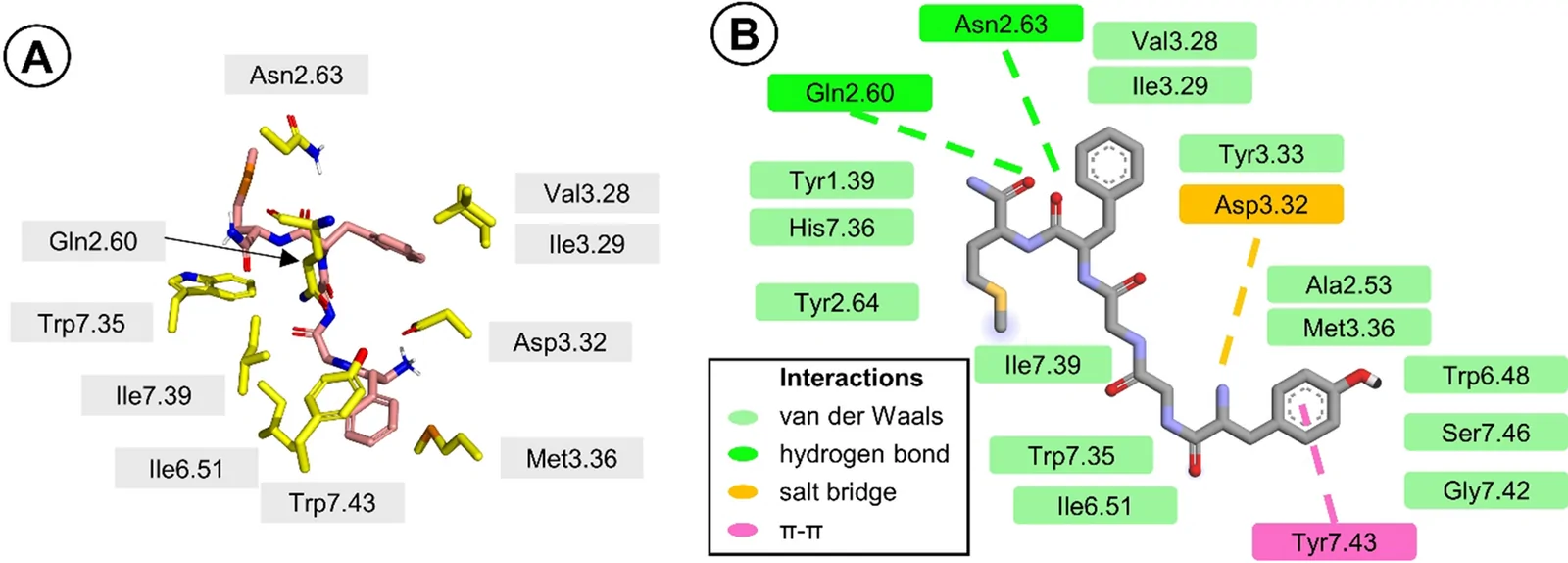
Binding mode of **[Met**^**5**^**]enk-NH**_**2**_ to MOR. (**a**) The molecule in the receptor binding site. Only selected residues of the receptor are displayed (yellow). The ligand is shown as salmon sticks. Hydrogen atoms’ display is suppressed, except in selected cases. (**b**) Interaction diagram. The interacting residues are colored according to the legend in the Figure

The linear **[Met**^**5**^**]enk-NH**_**2**_ is predicted to insert its N-terminus deep in the binding pocket (Fig. 6). Protonated amine of Tyr^1^ forms a salt bridge to Asp3.32 and additionally, it is located relatively close to the aromatic ring of Tyr3.33. The aromatic ring of Tyr^1^ is wedged between Tyr7.43 and Met3.36 side chains and π-stacks with the aromatic ring of the former. Other receptor residues that contact Tyr^1^ by van der Waals interactions include Ala2.53, Trp6.48, Ser7.46, and Gly7.42. The middle fragment of the peptide (-Gly^2^-Gly^3^-) interacts with the side chains of Ile6.51, Trp7.35, and Ile7.39 (van der Waals interactions). The aromatic ring of Phe^4^ is inserted between Asp3.32 and Ile3.29 side chains and in proximity of Val3.28 side chain. The carbonyl oxygen of Phe^4^ is H-bonded to side chain amide hydrogen of Asn2.63. Met^5^ side chain approaches Tyr2.64. C-terminal carbonyl oxygen of the peptide H bonds to side chain amide hydrogen of Gln2.60 and this fragment is flanked by the side chains of His7.36 and Tyr1.39.

This predicted binding orientation for the linear **[Met**^**5**^**]enk-NH**_**2**_ is in the main features similar to that found by **DAMGO** in the experimental structure (their comparison is given in Fig. S1 in Supplementary Information). The differences include orientation of Tyr^1^ aromatic ring (protruding deeper in the case of **[Met**^**5**^**]enk-NH**_**2**_) and somewhat displaced position of Phe^4^ aromatic ring.

**Fig. 7:**
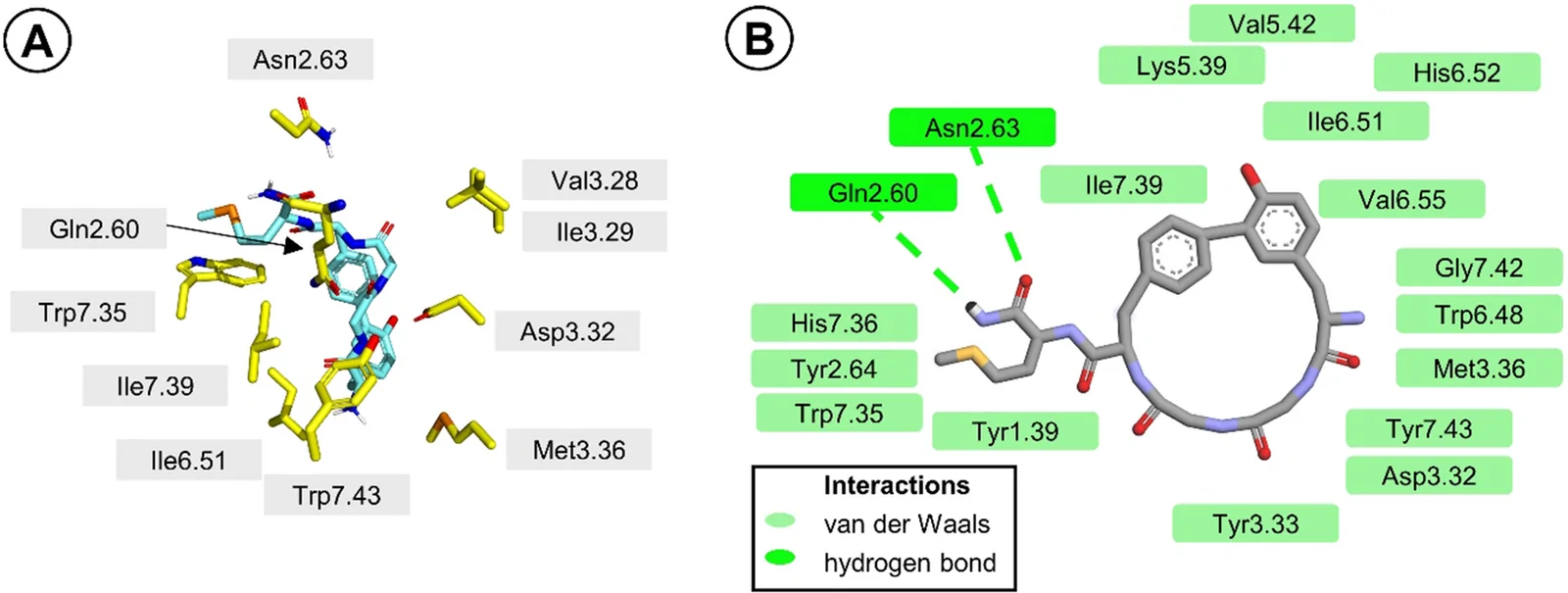
Binding mode of compound **c-(Tyr-*m*-Phe-*p*)-M-NH**_**2**_ to MOR. (**a**) The molecule in the receptor binding site. Only selected residues of the receptor are displayed (yellow). The ligand is shown as salmon sticks. Hydrogen atoms’ display is suppressed, except in selected cases. (**b**) Interaction diagram. The interacting residues are colored according to the legend in the Figure

The predicted binding mode of **c-(Tyr-*m*-Phe-*p*)-M-NH**_**2**_ is shown in Fig. 7. Most strikingly, while the N-terminal portion of the biaryl peptide is directed to the bottom binding site, the protonated amine does not form interaction to Asp3.32 but is placed in the vicinity of Met3.36, Trp6.48, Ile6.51, His6.52, and Gly7.42. Tyr^1^ aromatic ring interacts with the side chain of Val6.55 (van der Waals interactions). The other ring of the biaryl fragment lies by Lys6.58 and Trp7.35. The -Gly^2^-Gly^3^- backbone locates close to the side chains of Gln2.60, Ile3.29, Asp3.32, and Tyr7.43. Met^5^ side chain is inserted between Tyr1.39, Tyr2.64, Trp7.35, and His7.36 side chains. The only two H bonds in the predicted binding pose of the biaryl analog are located in the C-terminal part of the peptide. They are the interaction between the peptide’s amide hydrogen and the backbone carbonyl oxygen of Gln2.60 and between the peptide’s amide oxygen and the side chain carbonyl oxygen of Asn2.63.

This binding pose would be intuitively expected to be of rather limited affinity, in particular due to lack of the amine-Asp3.32 interaction which is usually assumed to be prerequisite for high-affinity binding to MOR (Li et al. 1999) although exceptions are known (De Marco and Gentilucci 2017). Furthermore, it is to be noted that the binding mode predicted for the biaryl **c-(Tyr-*m*-Phe-*p*)-M-NH**_**2**_ is clearly different than that predicted for the linear analog (comparison given in Fig. S2) or that obtained experimentally for **DAMGO**. The docking results correspond to low MOR affinity of **c-(Tyr-*m*-Phe-*p*)-M-NH**_**2**_ and suggest that the studied family of biaryl enkephalin analogs does not mimick the bioactive conformation of enkephalins. It is consistent with the hypothesis that the aromatic rings in the biologically active conformation of enkephalin should be located at a similar distance from each other like the tyramine moiety and atoms C-5 and C-6 of the C-ring of morphine as proposed by Gorin and Marshall (Gorin and Marshall 1977).

## Conclusion

We obtained 10 cyclic biaryl analogs of enkephalins using the Miyaura–Suzuki approach. The peptides were not easy to synthesize due to a large steric strain present in their cyclic parts. The novel compounds were studied by CD and NMR. The CD studies did not bring any essential information on their conformation due to their structural elements which make analysis of the CD spectra difficult. Much more useful in this case were the NMR investigations which allowed detailed determination of conformations of the peptides studied. The NMR-derived structures were obtained from the XPlor calculations. Because we were not sure if the XPlor package is well adapted for calculations on such a kind of compounds, we confronted the XPlor results with quantum chemical calculations on one of the peptide analogs. A satisfactory agreement was found between the results obtained by the two methods. The NMR studies showed that the peptides may adopt the conformations which can be described as a type IV β-turn. No intramolecular hydrogen bonds were found for all the peptides. An interesting feature of their conformations is a distinct pyramidization of the aromatic carbon atoms forming the biaryl bridge. Seven of the peptides were checked in vitro for their affinity for the µ-opioid receptor. Unfortunately, it was found that none of them exhibits a significant MOR affinity with the best analog having IC_50_ as low as 1372.5 nM which is a several-100-fold worse value that those found for linear enkephalins. This results most probably from the direct bond between two aromatic rings as it has been postulated earlier that these rings should be located at a similar distance from each other like the corresponding structural elements in morphine.

This work confirms that the Miyaura–Suzuki method is a very effective and versatile way of preparation of cyclic biaryl peptides. This kind of peptide cyclization can be very attractive and promising in the case of other biologically active peptides which contain aromatic amino acid residues, allowing diverse modifications of the parent molecules.

## Supplementary Information

Below is the link to the electronic supplementary material.

Supplementary file1 (ZIP 1030 KB)

## Data Availability

The data presented in this study are available upon request from the corresponding authors.
