# Supplementary material for: Applications of biaryl cyclization in the synthesis of cyclic enkephalin analogs with a highly restricted flexibility
Source: Amino Acids. 2024 Mar 1;56(1):18. doi: 10.1007/s00726-023-03371-5 (PMC10907494; doi:10.1007/s00726-023-03371-5)
Supplement: Supplementary file 1 — Supplementary file1 (ZIP 1030 KB) [file 726_2023_3371_MOESM1_ESM.zip › SI/Supplementary_Inforamtion_corrected.docx]

**Supplementary Information**for

*Applications of biaryl cyclization in the synthesis of cyclic enkephalin analogs with a highly restricted flexibility*

**Maria Różanowska^1*^, Gabriela Szczupaj^2^, Michał Nowakowski^2^, Priyadharshni Rajagopal^3^, Piotr F.J. Lipiński^4^, Joanna Matalińska^4^, Aleksandra Misicka^4,5^, Marek Lisowski^1^, Łukasz Jaremko^3^, Mariusz Jaremko^3*^**

1 Faculty of Chemistry, University of Wrocław, Wrocław, Poland;

2 Faculty of Chemistry, Biological and Chemical Research Centre, University of Warsaw, Warsaw, Poland

3 Division of Biological and Environmental Sciences and Engineering (BESE), King Abdullah University of Science and Technology (KAUST), Thuwal, Saudi Arabia

4 Department of Neuropeptides, Mossakowski Medical Research Institute, Polish Academy of Sciences, Warsaw, Poland

5 Faculty of Chemistry, University of Warsaw, Warsaw, Poland

* Correspondence: [maria.rozanowska2@uwr.edu.pl](mailto:maria.rozanowska2@uwr.edu.pl) [mariusz.jaremko@kaust.edu.sa](mailto:mariusz.jaremko@kaust.edu.sa)

List of contents

1. **Table S-1.** Interresidue nontrivial NOE contacts in the peptides studied**……………...……...……….2**

2. **Table S-2.** Comparison of backbone torsion angles of different **c-(Tyr-*m*-Phe-*p*)-M-NH_2_**
 structures**………………………………………………………….……………….………4**

3. **Table S-3.** Results of initial screening for affinity towards the μ-opioid receptor**……..…..…………..4**

4. **Fig. S-1.** Comparison of MOR binding modes of **[Met^5^]enk-NH_2_** and **DAMGO………..….……..…5**

5**. Fig. S-2.** Comparison of MOR binding modes of **[Met^5^]enk-NH_2_** and **c-(Tyr-*m*-Phe-*p*)-M-NH_2_.......5**

6. **Reagents used for syntheses and purification of peptides…….……………….……………………5**

7. **Syntheses of aromatic amino acid derivatives, cyclic and linear peptides………………………....6**

8. **Table S-4.** Parameters of NMR measurements**……………………………………………….…....…12**

9. **Listing S-1.** Atomic coordinates of **c-(Tyr-*m*-Phe-*p*)-M-NH_2_** molecule, lowest-energy geometry
 calculated by XPLOR**………………………………………...………………….……...14**

10**. Listing S-2.** Atomic coordinates of **c-(Tyr-*m*-Phe-*p*)-M-NH_2_** molecule optimized at the
 B3LYP/6-31G(d,p) level with PCM solvent model (water)**………….… ……………15**

11. **Table S-5.** Docking scores for top MOR docking poses of **[Met^5^]enk-NH_2_………….……..……...17**

12. **Table S-6.** Docking scores for top MOR docking poses of **c-(Tyr-*m*-Phe-*p*)-M-NH_2_…….........….17**

.

**Table S-1**. Interresidue nontrivial NOE contacts in the peptides studied detected from the ROESY spectra in H_2_O/D_2_O (9:1, v/v).

| **c-(Tyr-*m*-Phe-*p*)-L-NH_2_** | | |
| --- | --- | --- |
| Tyr^1^ C^δ^H1 ↔ Leu^5^ C^δ2^H^a^ | Gly^2^ NH ↔ Tyr^1^ C^δ^H1 | Phe^4^ C^δ^H^b^ ↔ Leu^5^ NH |
| Tyr^1^ C^α^H ↔ Phe^4^ C^δ^H^b^ | Gly^3^ C^α^H1 ↔ Phe^4^ NH | Phe^4^ C^ε^H^b^ ↔ Gly^2^ NH |
| Tyr^1^ C^δ^H1 ↔ Gly^3^ C^α^H1 | Gly^3^ C^α^H1 ↔ Phe^4^ C^δ^H^b^ | Phe^4^ NH ↔ Leu^5^ NH |
| Tyr^1^ C^β^H1 ↔ Gly^2^ NH | Gly^3^ C^α^H1 ↔ Phe^4^ C^ε^H^b^ | Leu^5^ C^α^H ↔ Gly^2^ C^α^H2 |
| Tyr^1^ C^δ^H2 ↔ Gly^2^ C^α^H1 | Gly^3^ C^α^H2 ↔ Gly^2^ C^α^H1 | Leu^5^ NH ↔ Gly^2^ C^α^H2 |
| Tyr^1^ C^δ^H1 ↔ Gly^2^ C^α^H1 | Gly^3^ C^α^H2 ↔ Phe^4^ NH | Leu^5^ C^α^H ↔ Phe^4^ C^ε^H^b^ |
| Gly^2^ C^α^H1 ↔ Leu^5^ NH | Phe^4^ C^α^H ↔ Leu^5^ NH | Leu^5^ C^γ^H ↔ Gly^2^ C^α^H1 |
| Gly^2^ C^α^H1 ↔ Leu^5^ C^α^H | Phe^4^ C^β^H1 ↔ Leu^5^ NH | Leu^5^ C^δ2^H^a^ ↔ Gly^2^ C^α^H1 |
| Gly^2^ C^α^H1 ↔ Gly^3^ NH | Phe^4^ C^β^H2 ↔ Leu^5^ NH | Leu^5^ C^δ1^H^a^ ↔ Gly^2^ C^α^H1 |
| Gly^2^ C^α^H2 ↔ Gly^3^ NH | Phe^4^ C^δ^H^b^ ↔ Gly^2^ C^α^H1 | Phe^4^ NH ↔ Gly^2^ C^α^H2 |
| **c-(Tyr-*m*-Phe-*m*)-L-NH_2_** | | |
| Tyr^1^ C^δ^H2 ↔ Phe^4^ C^δ^H1 | Tyr^1^ C^δ^H2 ↔ Gly^2^ NH | Gly^3^ C^α^H2 ↔ Gly^2^ C^α^H2 |
| Tyr^1^ C^β^H2 ↔ Phe^4^ C^β^H1 | Tyr^1^ C^δ^H2 ↔ Gly^2^ C^α^H2 | Phe^4^ C^α^H ↔ Leu^5^ NH |
| Tyr^1^ C^δ^H2 ↔ Phe^4^ C^ε^H2 | Gly^2^ NH ↔ Tyr^1^ C^α^H | Phe^4^ NH ↔ Leu^5^ NH |
| Tyr^1^ C^β^H1 ↔ Glt^3^ C^α^H2 | Gly^2^ C^α^H1 ↔ Gly^3^ NH | Phe^4^ C^β^H1 ↔ Gly^3^ C^α^H2 |
| Gly^3^ NH ↔ Leu^5^ C^α^H | Gly^2^ C^α^H2 ↔ Gly^3^ NH | Phe^4^ C^β^H2 ↔ Leu^5^ NH |
| Gly^2^ C^α^H2 ↔ Leu^5^ C^α^H | Gly^2^ C^α^H2 ↔ Gly^3^ C^α^H1 | Leu^5^ NH ↔ Phe^4^ C^β^H1 |
| Gly^3^ C^α^H2 ↔ Phe^4^ NH | Gly^3^ NH ↔ Phe^4^ NH | Leu^5^ C^α^H ↔ Phe^4^ NH |
| Phe^4^ C^β^H1 ↔ Tyr^1^ C^β^H1 | Gly^3^ C^α^H1 ↔ Phe^4^ NH | Leu^5^ C^α^H ↔ Phe^4^ C^δ^H1 |
| Tyr^1^ C^β^H1 ↔ Gly^2^ NH |  |  |
| **c-(Tyr-*m*-Phe-*p*)-M-NH_2_** | | |
| Tyr^1^ C^α^H ↔ Met^5^ C^γ^H2 | Phe^4^ C^δ^H^b^ ↔ Tyr^1^ C^β^H1 | Gly^2^ C^α^H2 ↔ Met^5^ C^β^H1 |
| Tyr^1^ C^δ^H1 ↔ Met^5^ NH | Phe^4^ C^δ^H^b^ ↔ Tyr^1^ C^β^H2 | Gly^2^ C^α^H1 ↔ Gly^3^ NH |
| Tyr^1^ C^δ^H1 ↔ Met^5^ C^α^H | Gly^2^ NH ↔ Met^5^ C^γ^H2 | Gly^2^ C^α^H2 ↔ Tyr^1^ C^δ^H2 |
| Tyr^1^ C^ε^H2 ↔ Met^5^ C^γ^H2 | Phe^4^ NH ↔ Gly^3^ C^α^H1 | Gly^2^ C^α^H2 ↔ Gly^3^ NH |
| Tyr^1^ C^δ^H1 ↔ Phe^4^ C^β^H1 | Phe^4^ NH ↔ Met^5^ NH | Phe^4^ C^β^H2 ↔ Met^5^ NH |
| Tyr^1^ C^δ^H2 ↔ Gly^3^ NH | Gly^2^ C^α^H1 ↔ Phe^4^ C^β^H1 | Gly^3^ NH ↔ Phe^4^ NH |
| Tyr^1^ C^δ^H2 ↔ Gly^2^ NH | Gly^2^ C^α^H1 ↔ Phe^4^ NH | Gly^3^ C^α^H1 ↔ Gly^2^ C^α^H1 |
| Tyr^1^ C^δ^H2 ↔ Gly^3^ C^α^H1 | Gly^2^ C^α^H1 ↔ Met^5^ NH | Gly^3^ C^α^H1 ↔ Phe^4^ NH |
| Tyr^1^ C^δ^H2 ↔ Gly^3^ C^α^H2 | Gly^2^ C^α^H1 ↔ Met^5^ C^α^H | Gly^3^ C^α^H2 ↔ Gly^2^ C^α^H1 |
| Tyr^1^ C^β^H1 ↔ Gly^2^ NH | Phe^4^ C^β^H1 ↔ Met^5^ NH | Gly^3^ C^α^H2 ↔ Phe^4^ NH |
| Tyr^1^ C^β^H1 ↔ Gly^2^ C^α^H1 | Phe^4^ C^β^H1 ↔ Met^5^ NH1^c^ | Phe^4^ C^δ^H^b^ ↔ Gly^2^ C^α^H1 |
| Tyr^1^ C^β^H2 ↔ Gly^2^ C^α^H1 | Gly^2^ C^α^H1 ↔ Met^5^ C^β^H1 | Phe^4^ C^δ^H^b^ ↔ Gly^3^ C^α^H1 |
| Gly^2^ NH ↔ Met^5^ C^α^H | Gly^2^ C^α^H1 ↔ Met^5^ C^β^H2 | Phe^4^ C^β^H2 ↔ Gly^2^ C^α^H1 |
| Phe^4^ NH ↔ Tyr^1^ C^α^H | Gly^2^ C^α^H1 ↔ Met^5^ C^γ^H2 | Phe^4^ C^δ^H^b^ ↔ Gly^3^ C^α^H2 |
| Met^5^ C^γ^H*1 ↔ Gly^2^ C^α^H1 |  |  |
| **c-(Tyr-*m*-Phe-*p*)-L-OH** | | |
| Tyr^1^ C^δ^H2 ↔ Gly^2^ NH | Gly^3^ C^α^H1 ↔ Phe^4^ NH | Phe^4^ NH ↔ Leu^5^ NH |
| Gly^2^ C^α^H1 ↔ Tyr^1^ C^δ^H2 | Gly^3^ C^α^H1 ↔ Tyr^1^ C^δ^H2 | Phe^4^ C^α^H ↔ Tyr^1^ C^δ^H1 |
| Gly^2^ C^α^H1 ↔ Tyr^1^ C^δ^H1 | Gly^3^ NH ↔ Gly^2^ C^α^H2 | Phe^4^ C^α^H ↔ Tyr^1^ C^ε^H1 |
| Gly^2^ C^α^H1 ↔ Gly^3^ NH | Leu^5^ C^γ^H ↔ Phe^4^ C^ε^H^b^ | Phe^4^ C^β^H1 ↔ Leu^5^ NH |
| Gly^3^ C^α^H1 ↔ Phe^4^ C^δ^H^b^ | Leu^5^ C^β^H^b^ ↔ Phe^4^ C^ε^H^b^ | Phe^4^ C^β^H2 ↔ Leu^5^ NH |
| Gly^3^ C^α^H2 ↔ Phe^4^ NH | Leu^5^ C^δ2^H^a^ ↔ Phe^4^ C^ε^H^b^ | Phe^4^ C^ε^H^b^ ↔ Leu^5^ NH |
| Gly^3^ C^α^H2 ↔ Phe^4^ C^δ^H^b^ | Tyr^1^ C^β^H1 ↔ Gly^2^ NH | Phe^4^ C^ε^H^b^ ↔ Leu^5^ C^δ1^H^a^ |
| **c-(Tyr-*m*-Tyr-*m*)-L-NH_2_** | | |
| Tyr^1^ C^δ^H2 ↔ Tyr^4^ C^ε^H | Tyr^4^ C^α^H ↔ Leu^5^ NH | Tyr^4^ C^β^H2 ↔ Leu^5^ NH |
| Tyr^1^ C^δ^H1 ↔ Tyr^4^ C^δ^H1 | Tyr^4^ C^α^H ↔ Leu^5^ C^γ^H | Gly^2^ C^α^H1 ↔ Gly^3^ NH |
| Tyr^1^ C^β^H1 ↔ Gly^2^ C^α^H2 | Tyr^4^ C^α^H ↔ Leu^5^ C^β^H^b^ | Gly^2^ C^α^H2 ↔ Gly^3^ NH |
| Tyr^1^ C^β^H1 ↔ Gly^3^ C^α^H1 | Tyr^4^ C^α^H ↔ Leu^5^ C^δ1^H^a^ | Gly^2^ C^α^H2 ↔ Gly^3^ C^α^H1 |
| Tyr^1^ C^β^H1 ↔ Gly^3^ C^α^H2 | Tyr^4^ C^α^H ↔ Leu^5^ C^δ2^H^a^ | Gly^3^ C^α^H1 ↔ Tyr^4^ NH |
| Tyr^4^ C^α^H ↔ Tyr^1^ C^δ^H2 | Tyr^4^ C^β^H1 ↔ Leu^5^ NH1^c^ | Gly^3^ C^α^H2 ↔ Gly^2^ C^α^H2 |
| Tyr^4^ C^β^H1 ↔ Gly^2^ C^α^H2 | Tyr^4^ C^β^H1 ↔ Gly^3^ C^α^H2 | Gly^3^ C^α^H2 ↔ Tyr^4^ NH |
| Tyr^4^ NH ↔ Gly^3^ C^α^H2 | Tyr^4^ C^β^H1 ↔ Leu^5^ NH |  |
| **c-(Phe-*p*-Tyr-*m*)-L-NH_2_** | | |
| Phe^1^ C^δ^H^b^ ↔ Leu^5^ NH | Gly^2^ C^α^H2 ↔ Tyr^4^ C^ε^H2 | Phe^1^ C^β^H1 ↔ Gly^2^ C^α^H2 |
| Phe^1^ C^δ^H^b^ ↔ Tyr^4^ NH | Gly^2^ C^α^H1 ↔ Gly^3^ NH | Phe^1^ C^β^H2 ↔ Gly^2^ C^α^H1 |
| Phe^1^ C^ε^H^b^ ↔ Tyr^4^ NH | Gly^2^ C^α^H2 ↔ Phe^1^ C^ε^H^b^ | Tyr^4^ NH ↔ Leu^5^ NH |
| Phe^1^ C^ε^H^b^ ↔ Tyr^4^ C^ε^H2 | Gly^2^ C^α^H1 ↔ Gly^3^ C^α^H2 | Tyr^4^ C^α^H ↔ Leu^5^ NH |
| Tyr^4^ C^δ^H2 ↔ Phe^1^ C^δ^H^b^ | Gly^2^ C^α^H2 ↔ Phe^1^ C^δ^H^b^ | Tyr^4^ C^β^H1 ↔ Leu^5^ NH |
| Tyr^4^ C^δ^H2 ↔ Phe^1^ C^ε^H^b^ | Gly^2^ C^α^H2 ↔ Gly^3^ NH | Tyr^4^ C^β^H2 ↔ Leu^5^ NH |
| Tyr^4^ C^ε^H2 ↔ Phe^1^ C^δ^H^b^ | Gly^3^ C^α^H1 ↔ Gly^2^ C^α^H2 | Tyr^4^ C^δ^H2 ↔ Leu^5^ NH |
| Phe^1^ C^β^H1 ↔ Gly^3^ C^α^H2 | Gly^3^ C^α^H1 ↔ Tyr^4^ C^ε^H2 | Tyr^4^ C^ε^H2 ↔ Leu^5^ NH |
| Phe^1^ C^ε^H^b^ ↔ Gly^3^ NH | Phe^1^ C^α^H ↔ Gly^2^ NH | Leu^5^ C^δ2^H^a^ ↔ Tyr^4^ C^ε^H2 |
| Phe^1^ C^ε^H^b^ ↔ Gly^3^ NH |  |  |
| **c-(Phe-*p*-Phe-*p*)-L-NH_2_** | | |
| Phe^1^ C^ε^H^b^ ↔ Gly^3^ NH | Gly^3^ NH ↔ Phe^4^ NH | Gly^3^ NH ↔ Gly^2^ C^α^H2 |
| Phe^1^ C^α^H ↔ Gly^2^ NH | Gly^2^ C^α^H2 ↔ Phe^1^ C^δ^H^b^ | Phe^4^ NH ↔ Phe^1^ C^δ^H^b^ |
| Phe^4^ C^α^H ↔ Leu^5^ NH | Gly^2^ C^α^H2 ↔ Phe^4^ C^β^H1 | Gly^3^ C^α^H1 ↔ Phe^4^ NH |
| Phe^4^ C^β^H1 ↔ Leu^5^ NH | Gly^2^ C^α^H1 ↔ Phe^1^ C^α^H | Gly^3^ C^α^H2 ↔ Phe^4^ NH |
| Phe^4^ C^β^H2 ↔ Leu^5^ NH | Gly^3^ NH ↔ Phe^1^ C^α^H | Phe^4^ NH ↔ Leu^5^ NH |
| Phe^1^ C^ε^H^b^ ↔ Phe^4^ C^ε^H^b^ | Gly^2^ NH ↔ Phe^1^ C^β^H2 | Leu^5^ C^α^H ↔ Phe^1^ C^ε^H^b^ |
| **c-(Phe-*m*-Phe-*p*)-L-NH_2_** | | |
| Phe^1^ C^α^H ↔ Gly^2^ NH | Gly^3^ NH ↔ Phe^4^ NH | Phe^4^ C^ε^H^b^ ↔ Gly^2^ C^α^H2 |
| Phe^1^ C^β^H1 ↔ Gly^2^ NH | Gly^3^ C^α^H1 ↔ Phe^4^ NH | Phe^1^ C^ζ^H ↔ Phe^4^ C^ε^H^b^ |
| Phe^1^ C^δ^H1 ↔ Gly^2^ NH | Gly^3^ C^α^H1 ↔ Phe^4^ C^δ^H^b^ | Phe^4^ NH ↔ Leu^5^ NH |
| Phe^1^ C^δ^H1 ↔ Gly^2^ C^α^H1 | Gly^3^ C^α^H1 ↔ Phe^4^ C^ε^H^b^ | Phe^4^ C^α^H ↔ Leu^5^ NH |
| Gly^2^ C^α^H1 ↔ Gly^3^ NH | Gly^3^ C^α^H2 ↔ Phe^4^ NH | Phe^4^ C^β^H1 ↔ Gly^3^ NH |
| Gly^2^ NH ↔ Phe^1^ C^β^H2 | Phe^4^ C^β^H1 ↔ Gly^2^ NH | Phe^4^ C^β^H1 ↔ Leu^5^ NH |
| Gly^2^ NH ↔ Gly^3^ C^α^H2 | Phe^4^ C^δ^H^b^ ↔ Gly^2^ C^α^H2 | Phe^4^ C^β^H2 ↔ Leu^5^ NH |
| Gly^3^ NH ↔ Gly^2^ C^α^H2 | Phe^4^ C^ε^H^b^ ↔ Gly^2^ C^α^H1 |  |
| **c-(Phe-*p*-Phe-*m*)-L-NH_2_** | | |
| Phe^1^ C^δ^H^b^ ↔ Gly^3^ C^α^H1 | Gly^2^ C^α^H1 ↔ Phe^1^ C^δ^H^b^ | Phe^4^ C^β^H2 ↔ Leu^5^ NH |
| Phe^1^ C^δ^H^b^ ↔ Gly^3^ C^α^H2 | Gly^2^ C^α^H1 ↔ Phe^1^ C^ε^H^b^ | Leu^5^ C^α^H ↔ Phe^4^ C^ε^H |
| Phe^1^ C^δ^H^b^ ↔ Gly^2^ C^α^H2 | Gly^2^ C^α^H1 ↔ Gly^3^ NH | Gly^3^ C^α^H1 ↔ Phe^4^ NH |
| Phe^1^ C^ε^H^b^ ↔ Gly^3^ C^α^H2 | Phe^4^ C^α^H ↔ Leu^5^ NH | Gly^3^ C^α^H2 ↔ Phe^4^ NH |
| Phe^1^ C^ε^H ^b^ ↔ Gly^2^ C^α^H2 | Phe^4^ C^β^H1 ↔ Leu^5^ NH |  |
| **c-(Phe-*m*-Phe-*m*)-L-NH_2_** | | |
| Phe^1^ C^α^H ↔ Leu^5^ C^β^H1 | Phe^1^ C^ε^H2 ↔ Leu^5^ C^δ1^H^a^ | Gly^2^ C^α^H1 ↔ Gly^3^ NH |
| Phe^1^ C^α^H ↔ Leu^5^ C^δ2^H^a^ | Phe^1^ C^ζ^H ↔ Leu^5^ NH1^c^ | Gly^3^ C^α^H1 ↔ Phe^4^ NH |
| Phe^1^ C^α^H ↔ Gly^2^ NH | Phe^1^ C^ζ^H ↔ Leu^5^ C^δ2^H^a^ | Gly^3^ C^α^H1 ↔ Phe^4^ C^δ^H2 |
| Gly^2^ C^α^H1 ↔ Phe^4^ NH | Phe^1^ C^δ^H2 ↔ Phe^4^ NH | Phe^4^ C^α^H ↔ Leu^5^ NH |
| Gly^2^ C^α^H2 ↔ Phe^4^ C^δ^H2 | Phe^1^ C^δ^H2 ↔ Phe^4^ C^δ^H2 | Phe^4^ C^β^H1 ↔ Leu^5^ NH1^c^ |
| Gly^3^ C^α^H2 ↔ Phe^4^ NH | Phe^1^ C^δ^H1 ↔ Phe^4^ C^β^H2 | Phe^4^ C^β^H1 ↔ Leu^5^ NH |
| Gly^3^ C^α^H2 ↔ Phe^4^ C^δ^H2 | Phe^1^ C^β^H1 ↔ Gly^2^ NH | Phe^4^ C^β^H2 ↔ Leu^5^ NH |
| Leu^5^ NH2^c^ ↔ Phe^4^ C^δ^H2 | Gly^2^ C^α^H2 ↔ Gly^3^ NH | Phe^4^ NH ↔ Gly^3^ NH |
| Leu^5^ C^α^H ↔ Phe^4^ C^δ^H2 | Gly^2^ C^α^H2 ↔ Phe^1^ C^δ^H1 | Phe^4^ NH ↔ Leu^5^ NH |

^a^ Averaged methyl protons on the C^δ1^ or C^δ2^ atoms of Leu^5^.
^b^ Determination of the proton number was not possible.
^c^ Protons of the C-terminal amide group.

**Table S-2.** Comparison of backbone torsion angles of different **c-(Tyr-*m*-Phe-*p*)-M-NH_2_** structures.

|  | Residue | | | | | | | | | |
| --- | --- | --- | --- | --- | --- | --- | --- | --- | --- | --- |
|  | 1 | | 2 | | 3 | | 4 | | 5 | |
|  | ϕ | ψ | ϕ | ψ | ϕ | ψ | ϕ | ψ | ϕ | ψ |
| **Average from XPLOR** | - | -125.8°  (±44.2°) | 94.6°  (±31.7°) | -20.3°  (±65.8°) | -13.1°  (±55.0°) | -11.8°  (±3.0°) | -157.0°  (±11.9°) | -62.6°  (±4.2°) | -43.5°  (±22.3°) | **-** |
| **Lowest-energy from XPLOR** |  | -148.1 | 103.9 | 21.3 | -52.2 | -9.1 | -148.6 | -66.2 | -61.4 | **-** |
| **Additional minimization at the B3LYP/6-31G(d,p) in water (PCM)** |  | 129.9 | -162.4 | 38.6 | -103.8 | 11.2 | -155.1 | -54.3 | -136.7 |  |

**Table S-3.** Results of initial screening for affinity towards the μ-opioid receptor.

| **Peptide** | **% of radioligand specific binding at** | | | | | | **estimated IC_50_** |
| --- | --- | --- | --- | --- | --- | --- | --- |
|  | **30 μM** | | **10 μM** | | **3 μM** | |  |
|  | **mean^a)^** | **S.D.^b)^** | **mean^a)^** | **S.D.^b)^** | **mean^a)^** | **S.D.^b)^** |  |
| **c-(Tyr-*m*-Phe-*p*)-L-NH_2_** | 77 | 4 | 78 | 11 | 99 | 16 | > 30 μM |
| **c-(Tyr-*m*-Phe-*p*)-M-NH_2_** | 12 | 4 | 34 | 4 | 49 | 4 | ~ 1 μM |
| **c-(Phe-*p*-Phe-*p*)-L-NH_2_** | 73 | 19 | 78 | 7 | 110 | 2 | > 30 μM |
| **c-(Tyr-*m*-Tyr-*m*)-L-NH_2_** | 117 | 1 | 102 | 7 | 87 | 9 | >> 30 μM |
| **c-(Phe-*p*-Tyr-*m*)-L-NH_2_** | 17^c)^ | 3 | 34^c)^ | 2 | 62^c)^ | 6 | 3 μM < IC_50_ < 10 μM |
| **c-(Tyr-*m*-Phe-*p*)-L-OH** | 36 | 6 | 60 | 8 | 88 | 20 | 10 μM < IC_50_ < 30 μM |
| **c-(Tyr-*m*-Phe-*m*)-L-NH_2_** | 12 | 3 | 28 | 21 | 64 | 38 | 3 μM < IC_50_ < 10 μM |
| **[Phe^1^,Tyr^4^,Leu^5^]enk-NH_2_** | 43 | 11 | 61 | 15 | 85 | 4 | 10 μM < IC_50_ < 30 μM |
| **[Phe^1^,Leu^5^]enk-NH_2_** | 5^c)^ | 1 | 13^c)^ | 8 | 26^c)^ | 7 | < 3 μM |

^a)^ mean of two independent experiments, if not stated otherwise

^b)^ standard deviation

^c)^ mean of two independent experiments


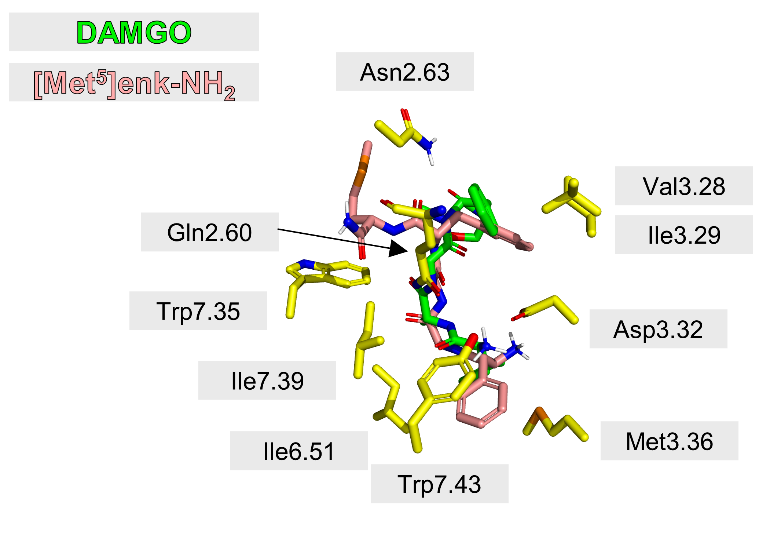


**Fig. S-1.** Comparison of MOR binding modes of **[Met^5^]enk-NH_2_** (salmon) and **DAMGO** (green). Both peptides are superposed. Only selected residues of the receptor are displayed (yellow). The ligand is shown as salmon sticks. Hydrogen atoms’ display is suppressed, except in selected cases.


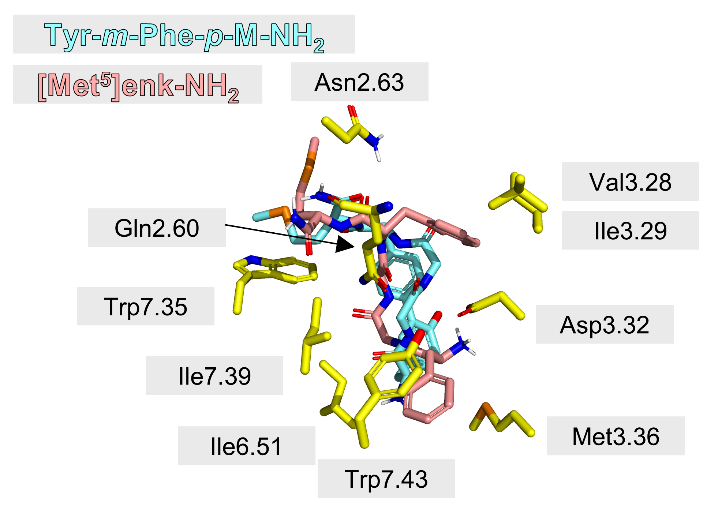


**Fig. S-2.** Comparison of MOR binding modes of **[Met^5^]enk-NH_2_** (salmon) and **c-(Tyr-*m*-Phe-*p*)-M-NH_2_** (aquamarine). Both peptides are superposed. Only selected residues of the receptor are displayed (yellow). The ligand is shown as salmon sticks. Hydrogen atoms’ display is suppressed, except in selected cases.

**Reagents used for syntheses and purification of peptides**

Standard Fmoc-amino acids derivatives were obtained from Novabiochem, Fmoc-*L*-Phe(3-I)-OH from Advachemlab, Fmoc-*L*-Phe(4-I)-OH from Fluorochem, and Boc-*L*- Phe(4-I)-OH from Sigma-Aldrich (Merck). Rink Amide MBHA resin, Fmoc-Leu-Wang resin, hexafluorophosphate 1-hydroxy-7-azabenzotriazole tetramethyl uranium (HATU), and N,N-diisopropylethylamine (DIPEA) were purchased from Novabiochem. 1,1′-Bis(diphenylphosphino)ferrocene]dichloropalladium(II), complex with dichloromethane (Pd(dppf)Cl_2_·CH_2_Cl_2_), and 1,1′-ferrocenediyl-bis(diphenylphosphine) (dppf) were obtained from Fluorochem. Di-*tert*-butyl dicarbonate (Boc_2_O), trityl chloride (Trt-Cl), triisopropylosilan (TIS), N,N-ethyldiisopropylamine (DIEA), silver sulfate, potassium acetate, potassium fluoride, potassium hydrogen sulfate, sodium hydrogen carbonate, potassium carbonate, lithium hydroxide, sodium hydroxide, and iodine were purchased from Sigma-Aldrich (Merck). Trifluoracetic acid (TFA) was obtained from IrisBiotech. Solvents for peptide and amino acid synthesis: N,N-dimethylformamide (DMF), dichloromethane (DCM), methanol (MeOH), diethyl ether (Et_2_O), dimethyl sulfoxide (DMSO), tetrahydrofuran (THF), and isopropanol were obtained from Sigma–Aldrich. Solvents for chromatography (acetonitrile and methanol, HPLC grade) were purchased from VWR chemicals. Solvents for MS and LC-MS (acetonitrile and methanol, LC-MS grade) were purchased from Riedel de Haën or J.T. Baker, formic acid (LC-MS grade) from Merck.

**Syntheses of aromatic amino acid derivatives and cyclic and linear peptides**

**Boc-Tyr(*^t^*Bu,3-I)-OMe.** Fmoc-*L*-Tyr(*^t^*Bu)-OH (5 g, 10.9 mmol) was dissolved in 10 ml of DMF and treated with 2.5 ml of piperidine. After 15 min about 100 ml of diethyl ether were added to the flask and obtained precipitate was filtered, washed a few times with diethyl ether, and dried. The precipitate obtained in the first stage of the reaction was transferred to a conical flask and 25 ml of dioxane/water (50:50) mixture and 1.1 eq of NaOH (0.48 g, 12 mmol) were added. After cooling the solution in the ice bath, Boc_2_O (1.1 eq, 12 mmol, 2.62 g) was added dropwise over about 1 hour (liquified in a water bath, 35°C). After Boc_2_O addition, the ice bath was removed and the solution was stirred for about 16 hours. The resulting solution was then carefully acidified with saturated aqueous KHSO_4_, extracted with ethyl acetate (2 × 50 mL), washed several times with water (100 ml) and brine (100 ml), dried over anhydrous sodium sulfate, and concentrated on a rotary evaporator to give a yellowish thick oil. Then 10 ml of DMF, K_2_CO_3_ (3 eq, 32.7 mmol, 4.56 g), and iodomethane (2.5 eq, 27 mmol, 1.69 ml) were added to the flask with the oil product. After two hours, the reaction was quenched by adding 100 ml of water and the reaction mixture was extracted with ethyl acetate (2 × 30 ml). The combined organic layers were washed several times with water (60 ml) and brine (60 ml), dried over anhydrous MgSO_4_, and concentrated *in vacuo*. The obtained product crystalized on hexane addition. Boc-Tyr(*^t^*Bu)-OMe was obtained as colorless crystals (2.2 g, 6.3 mmol, 58%). 50 ml of methanol, Ag_2_SO_4_ (1.2 eq, 6.8 mmol, 2.1 g), I_2_ (1.4 eq, 8.0 mmol, 2.0 g), and NaHCO_3_ (1.1 eq, 6.3 mmol, 0.53 g ) were placed in a conical flask. After 5 min stirring, Boc-Tyr(*^t^*Bu)-OMe (2.0 g, 5.7 mmol) dissolved in 25 ml of methanol was added. After 2 hours (the color of the solution changed from brown-orange to light yellow), the reaction was stopped by adding a saturated solution of sodium thiosulfate. The solution was evaporated and the product was dissolved in ethyl acetate and filtered through celite rinsed later with additional ethyl acetate. The product dissolved in ethyl acetate was transferred to a separating funnel, and rinsed with sodium thiosulfate solution (50 ml), several times with water (50 ml) and brine (50 ml), and then dried over anhydrous magnesium sulfate. After concentrating on a rotary evaporator, the crude product was purified by a column chromatography on a silica gel (eluent: hexane/ethyl acetate 2:1). The clean product was obtained as a colorless oil (2.1 g, 4.4 mmol, 78%).

ESI-MS: *m/z* ([M+Na]^+^) found 500.089, calcd. for C_19_H_28_INNaO_5_ 500.089.

^1^H NMR (500 MHz, CDCN_3_) δ 1.35 (s, 9 H), 1.42 (s 9H), 2.80 (m, 1 H), 3.02 (m, 1H), 3.6 (s, 3H), 4.33 (m, 1H), 5.54 (d, J=7.3 Hz, 1H), 7.07 (d, J=8.3 Hz, 1H), 7.12 ( dd, J=8.3 Hz, 2.2 Hz, 1H), 7.64 (s, 1H); ^13^C NMR (500 MHz, CDCN_3_) δ 28.52, 29.49, 37.02, 52.73, 55.76, 82.01, 94.88, 122.35, 131.09, 134.74, 135.22, 140.99, 156.01.

**Boc-Tyr(*^t^*Bu,3-I)-OH.** Obtained Boc-Tyr(*^t^*Bu,3-I)-OMe (0.95g, 2.0 mmol) was dissolved in 10 ml of methanol and 0.24 g of LiOH (5 eq, 10 mmol, 0.24 g) dissolved in 5 ml of water was added. After about 18 hours, the solution was concentrated and water was added to the flask. Obtained solution was carefully acidified by adding saturated KHSO_4_ solution and extracted into ethyl acetate (2 × 20 ml). The combined organic layers were washed with water (40 ml) and brine (40 ml), dried over anhydrous magnesium sulfate, and concentrated. The product was obtained as a colorless oil (0.83 g, 1.8 mmol, 89%).

ESI-MS: *m/z* ([M+Na]^+^) found 486.074, calcd. for C_18_H_26_INNaO_5_ 486.074.

^1^H NMR (500 MHz, CDCN_3_) δ 1.35 (s, 9H), 1.42 (s 9 H), 2.80 (m, 1 H), 3.07 (m, 1 H), 4.30 (m, 1H), 5.50 (d, J=7.7 Hz, 1H), 7.07 (d, J=8.3 Hz, 1H), 7.15 ( dd, J=8.3 Hz, 2.2 Hz, 1H), 7.64 (s, 1H); 9.41 (s, 1H).

**Fmoc-Tyr(*^t^*Bu,3-I)-OH.** Fmoc-Tyr(*^t^*Bu)-OH (2 g, 4,35 mmol) was dissolved in DMF and K_2_CO_3_ (3 eq, 1.8 g, 13.1 mmol) and iodomethane (2.5 eq, 10.8 mmol, 0.68 ml) were added to the flask. After four hours, the reaction was quenched by adding 50 ml of water and extracted with ethyl acetate (2 × 30 ml). The combined organic layers were washed several times with water (60 ml), brine (60 ml), dried over anhydrous MgSO_4_ and concentrated *in vacuo*. The obtained product crystalized from the ethyl acetate/hexane mixture. Product was filtered under reduced pressure, washed with hexane, and dried to obtain colorless crystals (1.99 g, 4.2 mmol, 96.6%).

20 ml of methanol, Ag_2_SO_4_ (1.2 eq, 1.57 g, 5.0 mmol), I_2_ (1.5 eq, 6.3 mmol, 4.0 g), and NaHCO_3_ (1.1 eq, 0.39 g, 4.6 mmol) were placed in a conical flask. After stirring for 5 min, Fmoc-Tyr(*^t^*Bu)-OMe (1.99g, 4.2 mmol) dissolved in 50 ml of methanol was added. After 8 hours (the color of the solution changed from brown-orange to light yellow), the reaction was stopped by adding a saturated solution of sodium thiosulfate. Methanol was evaporated and the product was dissolved in ethyl acetate (30 ml) and filtered through celite which was rinsed afterwards with additional ethyl acetate. The product dissolved in ethyl acetate was transferred to a separating funnel and rinsed with a sodium thiosulfate solution (60 ml), several times with water (60 ml) and brine (60 ml), and then dried over anhydrous magnesium sulfate. After concentrating on a rotary evaporator, the crude product was purified by a column chromatography on a silica gel (eluent: hexane/ethyl acetate 1:2). Product was obtained as a white solid (2.0 g, 3.4 mmol, 80%).

Methyl ester hydrolysis was performed in 0.8 M CaCl_2_ solution in isopropanol/THF/water (1:1:1 v/v/v) with NaOH (1.2 eq, 4.0 mmol, 0.16 g) for 8 h at rt. Obtained solution was concentrated, acidified with 1M KHSO_4_ solution, and extracted with ethyl acetate (3 × 30 ml). Combined organic layers were washed with KHSO_4_ solution, water (90 ml), and brine (90 ml), dried over magnesium sulfate, and evaporated. The product was obtained as a colorless oil (1.67 g, 2.9 mmol, 85%).

ESI-MS: *m/z* ([M+Na]^+^) found 608.091, calcd. for C_28_H_28_INNaO_5_608.690.

^1^H NMR (500 MHz, CDCl_3_) δ 1.44 (s, 9H), 3.01 (m, 1H), 3.12 (m, 1H), 4.22 (t, J=6,9, 1H), 4.37 (m,1H), 4.44 (m,1H), 4.51 (s, 1H), 4.66 (d, J=6.7 Hz, 1H), 5.22 (d, j=7.6 Hz, 1H), 6.99 (m, 2H), 7.31 (m, 2H), 7.40 (t, J=7,4 Hz, 2H), 7.57 (t, J=6.5 Hz, 2H), 7.63 (s, 1H), 7.76 (d, J=7.76 Hz, 2H).

**Boc-Tyr(*^t^*Bu,3-Bpin)-OH.** Boc-Tyr(*^t^*Bu,3-I)-OMe (1 g, 2.1 mmol, 1 eq) was dissolved in degassed DMSO (20 ml) and B_2_pin_2_ (1.5 eq, 0.80 g, 3.1 mmol), KOAc (3 eq, 0.62 g, 6.3 mmol), and PdCl_2_(dppf)•CH_2_Cl_2_ (0.1eq, 0.16g, 0.2 mmol) were added. The solution was stirred under nitrogen at 80°C overnight. Then water was added (50 ml) and the product was extracted to diethyl ether (3×50 ml). Combined organic layers were washed a few times with water (150 ml) and brine (150 ml). After concentration, the residue was dissolved in ethyl acetate (20 ml) and filtered through a pad of celite and silica rinsed later with additional ethyl acetate. Due to the instability of the obtained boronate amino acid on silica, the product was purified on a short silica column with hexane/ethyl acetate (1:1) as an eluent. Fractions with the product were evaporated *in vacuo*, giving a colorless oil (0.61 g, 61%). The obtained product (0.5 g, 1.1 mmol) was dissolved in 10 ml of methanol and LiOH (5 eq, 5.5 mmol, 0.13g) dissolved in 5 ml of water was added. After about 18 hours, the solution was concentrated and water was added to the flask. The solution was carefully acidified by adding saturated KHSO_4_ solution and extracted with ethyl acetate (2 × 20 ml). The combined organic layers were washed with water (40 ml), brine (40 ml), dried over anhydrous magnesium sulfate, and concentrated. The product was obtained as a colorless oil (0.42g, 0.96 mmol, 87%).

ESI-MS: *m/z* ([M+Na]^+^) found 486.266, calcd. for C_24_H_38_BNNaO_7_ 486.262.

^1^H NMR (500 MHz, CDCN_3_) δ 1.51 (s, 9H), 1.51 (s, 12H), 3.03 (m, 1H), 3.28 (m, 1H), 4.48 (m, 1H), 5.65 (d, J=7.5 Hz, 1H), 7.17 (d, J=8.3 Hz, 1H), 7.39 ( dd, J=8.4 Hz, 2,5 Hz, 1H), 7.58 (s, 1H), 7.77 (s, 1H).

**Boc-Phe(4-Bpin)-OH.** To the solution of Boc-Phe(4-I)-OMe (1.0 g, 2.47 mmol) dissolved in dry and degassed DMSO (25 ml), bis(pinacolato)diboron (4.94 mmol, 2 eq, 1.25 g), dried potassium acetate (3.5 eq, 8.65 mmol), and 1,1’-bis(diphenylphosphino)ferrocene dichloropalladium (complex with dichloromethane) (0.1 eq, 0.25 mmol, 0.2 g) were added. The mixture was degassed on a sonic bath, saturated with nitrogen 3 more times, and heated in oil bath at 80°C overnight. Then water was added and product was extracted with diethyl ether (3 × 50 ml). Combined organic layers where washed a few times with water (150 ml) and brine (150 ml). After evaporation, the residue was dissolved in ethyl acetate (20 ml) and filtered through a pad of celite and silica rinsed later with additional ethyl acetate. Due to instability of the obtained boronate amino acid on silica, the product was purified on a short silica column, using hexane/ethyl acetate (1:1). Fractions with the product were concentrated *in vacuo* and a colorless oil was obtained (0.4 g, 0.98 mmol, 40%).

Obtained Boc-Phe(4-Bpin)-OMe was dissolved in 10 ml of methanol and 0.24 g of LiOH (5 eq, 10 mmol, 0.24 g) dissolved in 5 ml of water was added. After about 18 hours, the solution was concentrated and water was added to the flask. The solution was carefully acidified by adding saturated KHSO_4_ solution and extracted with ethyl acetate (2 × 20 ml). The combined organic layers were washed with water (40 ml), brine, dried over anhydrous magnesium sulfate, and concentrated. The product was obtained as
a colorless oil - 0,83 g (0.83 g, 1.8 mmol, 89%).

ESI-MS: *m/z* ([M+Na]^+^) found 414.203, calcd. for C_20_H_30B_NNaO_6_ 414.205.

^1^H NMR (500 MHz, CDCN_3_) δ 1.31 (s, 12H), 1.34 (s, 9H), 3.04 (m, 2H), 4.33 (m, 1H), 5.4 (d, J=6.6 Hz 1H), 7.24 and 7.64 (2 × 2H, AA’XX’ system, J=7.5 and 7.7 Hz); ^13^C NMR (500 MHz, CDCN_3_) δ 25.2, 28.49, 38.15, 52.73, 55.51, 80.05, 84.78, 129.85, 135.52, 141.75, 156.42, 173.53.

Cyclic peptides

**H-(cyclo-*m*,*p*)-[Tyr-Gly-Gly-Phe]-Leu-NH_2_.** Linear peptide Boc-Tyr(*^t^*Bu,3-I)-Gly-Gly-Phe(4-Bpin)-Leu-Rink amide MBHA was synthesized according to General Procedures A and B. The peptide was cyclized according to General Procedure C.

HPLC R_t_: 14.4 min, purity: 98.2%, yield 13.1%. ESI-MS: *m/z* ([M+Na]^+^) found 553.278, calcd. for C_28_H_36_N_6_O_6_ 553.277.

^1^H-NMR (700 MHz, H_2_O/D_2_O (9:1, v/v)), δ (ppm): 0.827, 0.857 (6H, d, d, 2 × C^δ^H_3_ Leu^5^), 1.569 (1H, m, C^γ^H Leu^5^), 1.542, 1.638 (2H, m, m, C^β^H_2_ Leu^5^), 2.755, 3.363 (2H, dd, dd, C^β^H_2_ Phe^4^), 3.071, 3.220 (2H, dd, dd, C^β^H_2_ Tyr^1^), 3.528, 4.083 (2H, dd, dd, C^α^H_2_ Gly^2^), 3.600, 3.689 (2H, dd, dd, C^α^H_2_ Gly^3^), 4.082 (1H, dd, C^α^H Tyr^1^), 4.307 (1H, m, C^α^H Leu^5^), 4.749 (1H, C^α^H Phe^4^), 6.897 (1H, d, C^ε^H Tyr^1^), 7.090, 7.325 (2H, dd?, s, C^δ^H Tyr^1^), 7.189 (2H, d, C^δ^H Phe^4^), 7.318 (1H, d, C^ε^H Phe^4^), 7.802 (1H, t, NH Gly^3^), 7.891 (1H, d, NH Phe^4^), 8.164 (1H, t, NH Gly^2^), 8.349 (1H, d, NH Leu^5^), n.a. (1H, NH Tyr^1^).

**H-(cyclo-*m*,*m*)-[Tyr-Gly-Gly-Phe]-Leu-NH_2_.** Linear peptide Boc-Tyr(*^t^*Bu,3-I)-Gly-Gly-Phe(4-Bpin)-Leu-Rink amide MBHA was synthesized according to General Procedures A and B. The peptide was cyclized according to General Procedure C.

HPLC R_t_: 15.7 min, purity 98.3%, yield 11.6%. ESI-MS: *m/z* ([M+Na]^+^) found 553.278, calcd. for C_28_H_36_N_6_O_6_ 553.277.

^1^H-NMR (700 MHz, H_2_O/D_2_O (9:1, v/v)), δ (ppm): 0.821, 0.851 (6H, d, d, 2 × C^δ^H_3_ Leu^5^), 1.563 (1H, m, C^γ^H Leu^5^), 1.543, 1.633 (2H, m, m, C^β^H_2_ Leu^5^), 2.939, 3.201 (2H, dd, dd, C^β^H_2_ Phe^4^), 3.049, 3.355 (2H, dd, dd, C^β^H_2_ Tyr^1^), 3.510, 3.869 (2H, dd, dd, C^α^H_2_ Gly^3^), 3.699, 4.298 (2H, dd, m?, C^α^H_2_ Gly^2^), 4.267 (1H, m, C^α^H Tyr^1^), 4.305 (1H, m, C^α^H Leu^5^), 4.793 (1H, C^α^H Phe^4^), 6.908 (1H, d, C^ε^H Tyr^1^), 7.005, 7.244 (2H, s, d, C^δ^H Phe^4^), 7.115, 7.215 (2H, dd?, d?, C^δ^H Tyr^1^), 7.325 (1H, d, C^ζ^H Phe^4^), 7.331 (1H, m?, C^ε^H Phe^4^), 7.937 (1H, d, NH Phe^4^), 8.100 (1H, t, NH Gly^3^), 8.248 (1H, dd?, NH Gly^2^), 8.411 (1H, d, NH Leu^5^), n.a. (1H, NH Tyr^1^).

**H-(cyclo-*m*,*p*)-[Tyr-Gly-Gly-Phe]-Leu-OH.** Linear peptide Boc-Tyr(*^t^*Bu,3-I)-Gly-Gly-Phe(4-Bpin)-Leu-Wang was synthesized according to General Procedures A and B. The peptide was cyclized according to General Procedure C.

HPLC R_t_: 16.2 min, purity: 100%, yield 6.7%. ESI-MS: *m/z* ([M+Na]^+^) found 554.262, calcd. for C_28_H_35_N_6_O_7_ 554.261.*

^1^H-NMR (700 MHz, H_2_O/D_2_O (9:1, v/v)), δ (ppm): 0.827, 0.850 (6H, d, d, 2 × C^δ^H_3_ Leu^5^), 1.588 (1H, m, C^γ^H Leu^5^), 1.592 (2H, m, C^β^H_2_ Leu^5^), 2.764, 3.370 (2H, dd, dd, C^β^H_2_ Phe^4^), 3.075, 3.219 (2H, dd, dd, C^β^H_2_ Tyr^1^), 3.527, 4.083 (2H, dd, m?, C^α^H_2_ Gly^2^), 3.600, 3.690 (2H, dd, dd, C^α^H_2_ Gly^3^), 4.086 (1H, m, C^α^H Tyr^1^), 4.285 (1H, m, C^α^H Leu^5^), 4.720 (1H, C^α^H Phe^4^), 6.905 (1H, d, C^ε^H Tyr^1^), 7.088, 7.327 (2H, d, s, C^δ^H Tyr^1^), 7.198 (2H, d, C^δ^H Phe^4^), 7.322 (1H, d, C^ε^H Phe^4^), 7.804 (1H, t, NH Gly^3^), 7.866 (1H, d, NH Phe^4^), 8.161 (1H, t, NH Gly^2^), 8.200 (1H, d, NH Leu^5^), n.a. (1H, NH Tyr^1^).

**H-(cyclo-*m*,*m*)-[Tyr-Gly-Gly-Tyr]-Leu-NH_2_.** Linear peptide Boc-Tyr(*^t^*Bu,3-Bpin)-Gly-Gly-Tyr(*^t^*Bu,3-I)-Leu-Rink MBHA was synthesized according to General Procedure A, with the use of Fmoc-Tyr(*^t^*Bu,3-I)-OH and Boc-Tyr(*^t^*Bu,3-Bpin)-OH. The peptide was cyclized according to General Procedure C.

HPLC R_t_: 11.9 min, purity: 98.8%, yield 15.1%. ESI-MS: *m/z* ([M+Na]^+^) found 569.273, calcd. for C_28_H_35_N_6_O_7_ 569.272.

^1^H-NMR (600 MHz, H_2_O/D_2_O (9:1, v/v)), δ (ppm): 0.814, 0.850 (6H, d, d, 2 × C^δ^H_3_ Leu^5^), 1.546 (1H, m, C^γ^H Leu^5^), 1.578, 1.658 (2H, m, m, C^β^H_2_ Leu^5^), 2.839, 3.136 (2H, dd, dd, C^β^H_2_ Tyr^4^), 3.089, 3.389 (2H, dd, dd, C^β^H_2_ Tyr^1^), 3.533, 3.897 (2H, dd, dd, C^α^H_2_ Gly^3^), 3.740, 4.381 (2H, dd, m, C^α^H_2_ Gly^2^), 4.288 (1H, m, C^α^H Leu^5^), 4.321 (1H, m, C^α^H Tyr^1^), 4.696 (1H, C^α^H Tyr^4^), 6.812, 7.161 (2H, d, d, C^δ^H Tyr^4^), 6.869 (1H, d, C^ε^H Tyr^1^), 6.876 (1H, d, C^ε^H Tyr^4^), 7.123, 7.222 (2H, dd, s, C^δ^H Tyr^1^), 7.992 (1H, d, NH Tyr^4^), 8.133 (1H, t, NH Gly^3^), 8.329 (1H, d, NH Leu^5^), 8.470 (1H, broad s, NH Gly^2^), n.a. (1H, NH Tyr^1^).

**H-(cyclo-*p*,*m*)-[Phe-Gly-Gly-Tyr]-Leu-NH_2_.** Linear peptide Boc-Phe(4-Bpin)-Gly-Gly-Tyr-(*^t^*Bu,3-I)-Leu-Rink MBHA was synthesized according to General Procedure A, with the use of Fmoc-Tyr(*^t^*Bu,3-I)-OH and Boc-Phe(4-Bpin)-OH. The peptide was cyclized according to General Procedure C.

HPLC R_t_: 16.0 min, purity: 98.1%, yield 13.8%. ESI-MS: *m/z* ([M+H]^+^) found 553.278, calcd. for C_28_H_35_N_6_O_7_ 553.277.

^1^H-NMR (600 MHz, H_2_O/D_2_O (9:1, v/v)), δ (ppm): 0.792, 0.828 (6H, d, d, 2 × C^δ^H_3_ Leu^5^), 1.490 (1H, m, C^γ^H Leu^5^), 1.535, 1.617 (2H, m, m, C^β^H_2_ Leu^5^), 2.864, 3.443 (2H, dd, dd, C^β^H_2_ Phe^1^), 2.974, 3.038 (2H, dd, dd, C^β^H_2_ Tyr^4^), 3.525, 3.674 (2H, dd, dd, C^α^H_2_ Gly^2^), 3.546, 3.853 (2H, dd, dd, C^α^H_2_ Gly^3^), 4.167 (1H, dd, C^α^H Phe^1^), 4.256 (1H, m, C^α^H Leu^5^), 4.471 (1H, m, C^α^H Tyr^4^), 6.908 (1H, d, C^ε^H Tyr^4^), 6.910, 7.050 (2H, d, dd?, C^δ^H Tyr^4^), 6.975 (1H, t, NH Gly^2^), 7.225 (2H, d, C^δ^H Phe^1^), 7.436 (1H, d, C^ε^H Phe^1^), 7.865 (1H, d, NH Tyr^4^), 7.895 (1H, t, NH Gly^3^), 8.295 (1H, d, NH Leu^5^), n.a. (1H, NH Phe^1^).

**H-(cyclo-*p*,*p*)-[Phe-Gly-Gly-Phe]-Leu-NH_2_.** Linear peptide Boc-Phe(4-I)-Gly-Gly-Phe(4-Bpin)-Leu-Rink amide MBHA was synthesized according to General Procedures A and B. The peptide was cyclized according to General Procedure C.

HPLC R_t_: 18.7 min, purity: 98.4%, yield 10.8%. ESI-MS: *m/z* ([M+H]^+^) found 537.283, calcd. for C_28_H_36_N_6_O_6_ 537.282.

^1^H-NMR (700 MHz, H_2_O/D_2_O (9:1, v/v)), ( (ppm): 0.809, 0.837 (6H, d, d, 2 × C^δ^H_3_ Leu^5^), 1.544 (1H, m, C^γ^H Leu^5^), 1.518, 1.609 (2H, m, m, C^β^H_2_ Leu^5^), 2.679, 3.367 (2H, dd, dd, C^β^H_2_ Phe^4^), 2.856, 3.427 (2H, dd, dd, C^β^H_2_ Phe^1^), 3.206, 3.561 (2H, dd, dd, C^α^H_2_ Gly^3^), 3.294, 3.379 (2H, dd, dd, C^α^H_2_ Gly^2^), 3.936 (1H, dd, C^α^H Phe^1^), 4.275 (1H, m, C^α^H Leu^5^), 4.580 (1H, C^α^H Phe^4^), 6.987 (1H, t, NH Gly^2^), 7.283 (2H, d, C^δ^H Phe^4^), 7.335 (2H, m?, C^δ^H Phe^1^), 7.341 (1H, m?, NH Gly^3^), 7.574 (2H, d, C^ε^H Phe^4^), 7.668 (2H, d, C^ε^H Phe^1^), 7.873 (1H, d, NH Phe^4^), 8.271 (1H, d, NH Leu^5^), n.a. (1H, NH Phe^1^).

**H-(cyclo-*m*,*p*)-[Phe-Gly-Gly-Phe]-Leu-NH_2_.** Linear peptide Boc-Phe(3-I)-Gly-Gly-Phe(4-Bpin)-Leu-Rink amide MBHA was synthesized according to General Procedures A and B. The peptide was cyclized according to General Procedure C.

HPLC R_t_: 18.5 min, purity: 97.0%, yield 13.1%. ESI-MS: *m/z* ([M+H]^+^) found 537.283, calcd. for C_28_H_36_N_6_O_6_ 537.282.

^1^H-NMR (700 MHz, H_2_O/D_2_O (9:1, v/v)), δ (ppm): 0.821, 0.855 (6H, d, d, 2 × C^δ^H_3_ Leu^5^), 1.554 (1H, m, C^γ^H Leu^5^), 1.539, 1.615 (2H, m, m, C^β^H_2_ Leu^5^), 2.704, 3.359 (2H, dd, dd, C^β^H_2_ Phe^4^), 3.176, 3.234 (2H, dd, dd, C^β^H_2_ Phe^1^), 3.514, 3.851 (2H, dd, dd, C^α^H_2_ Gly^3^), 3.728, 4.064 (2H, dd, dd, C^α^H_2_ Gly^2^), 3.998 (1H, dd, C^α^H Phe^1^), 4.304 (1H, m, C^α^H Leu^5^), 4.746 (1H, C^α^H Phe^4^), 7.229 (2H, d, C^δ^H Phe^4^), 7.255, 7.615 (2H, d, s, C^δ^H Phe^1^), 7.424 (1H, dd, C^ε^H Phe^1^), 7.441 (1H, d, C^ε^H Phe^4^), 7.576 (1H, d, C^ζ^H Phe^1^), 7.689 (1H, t, NH Gly^3^), 7.838 (1H, t, NH Gly^2^), 8.005 (1H, d, NH Phe^4^), 8.342 (1H, d, NH Leu^5^), n.a. (1H, NH Phe^1^).

**H-(cyclo-*p*,*m*)-[Phe-Gly-Gly-Phe]-Leu-NH_2_.** Linear peptide Boc-Phe(4-I)-Gly-Gly-Phe(3-Bpin)-Leu-Rink amide MBHA was synthesized according to General Procedures A and B. The peptide was cyclized according to General Procedure C.

HPLC R_t_: 20.4 min, purity: 97.8%, yield 9.5%, ESI-MS: *m/z* ([M+H]^+^) found 537.283, calcd. for C_28_H_36_N_6_O_6_ 537.282.

^1^H-NMR (950 MHz, H_2_O/D_2_O (9:1, v/v)), δ (ppm): 0.807, 0.844 (6H, d, d, 2 × C^δ^H_3_ Leu^5^), 1.542 (1H, m, C^γ^H Leu^5^), 1.527, 1.612 (2H, m, m, C^β^H_2_ Leu^5^), 2.838, 3.418 (2H, dd, dd, C^β^H_2_ Phe^1^), 3.034, 3.179 (2H, dd, dd, C^β^H_2_ Phe^4^), 3.505, 3.807 (2H, d?, d?, C^α^H_2_ Gly^3^), 3.540, 3.623 (2H, d?, d?, C^α^H_2_ Gly^2^), 4.066 (1H, dd, C^α^H Phe^1^), 4.283 (1H, m, C^α^H Leu^5^), 4.452 (1H, m, C^α^H Phe^4^), 6.880 (1H, broad s?, NH Gly^2^), 7.239, 7.243 (2H, s, d, C^δ^H Phe^4^), 7.243 (2H, d, C^δ^H Phe^1^), 7.413 (1H, dd, C^ε^H Phe^4^), 7.529 (1H, d, C^ε^H Phe^1^), 7.539 (1H, d, C^ζ^H Phe^4^), 7.757 (1H, broad s?, NH Gly^3^), 7.970 (1H, d, NH Phe^4^), 8.343 (1H, d, NH Leu^5^), n.a. (1H, NH Phe^1^).

**H-(cyclo-*m*,*m*)-[Phe-Gly-Gly-Phe]-Leu-NH_2_.** Linear peptide Boc-Phe(3-I)-Gly-Gly-Phe(3-Bpin)-Leu-Rink amide MBHA was synthesized according to General Procedures A and B. The peptide was cyclized according to General Procedure C.

HPLC R_t_: 19.0 min, purity: 97.6%, yield 8.8%, ESI-MS: *m/z* ([M+H]^+^) found 537.283, calcd. for C_28_H_36_N_6_O_6_ 537.282.

^1^H-NMR (700 MHz, H_2_O/D_2_O (9:1, v/v)), δ (ppm): 0.821, 0.852 (6H, d, d, 2 × C^δ^H_3_ Leu^5^), 1.584 (1H, m, C^γ^H Leu^5^), 1.552, 1.637 (2H, m, m, C^β^H_2_ Leu^5^), 2.970, 3.175 (2H, dd, dd, C^β^H_2_ Phe^4^), 3.133, 3.421 (2H, dd, dd, C^β^H_2_ Phe^1^), 3.506, 3.820 (2H, dd, dd, C^α^H_2_ Gly^3^), 3.675, 4.331 (2H, dd, dd, C^α^H_2_ Gly^2^), 4.273 (1H, dd, C^α^H Phe^1^), 4.309 (1H, m, C^α^H Leu^5^), 4.701 (1H, C^α^H Phe^4^), 7.161, 7.271 (2H, s, d, C^δ^H Phe^4^), 7.278, 7.470 (2H, d, s, C^δ^H Phe^1^), 7.383 (1H, t?, C^ε^H Phe^1^), 7.431 (1H, t?, C^ε^H Phe^4^), 7.449 (1H, d, C^ζ^H Phe^1^), 7.489 (1H, d, C^ζ^H Phe^4^), 7.944 (1H, d, NH Phe^4^), 8.064 (1H, t, NH Gly^3^), 8.137 (1H, broad s?, NH Gly^2^), 8.411 (1H, d, NH Leu^5^), n.a. (1H, NH Phe^1^).

**H-(cyclo-*m*,*p*)-[Tyr-Gly-Gly-Phe]-Met-NH_2_.** Linear peptide Boc-Tyr(*^t^*Bu,3-I)-Gly-Gly-Phe(4-Bpin)-Leu-Rink amide MBHA was synthesized according to General Procedures A and B. The peptide was cyclized according to General Procedure C.

HPLC R_t_: 13.15 min, purity: 97.2%, yield 11.6%, ESI-MS: *m/z* ([M+H]^+^) found 571.234, calcd. for C_28_H_36_N_6_O_6_ 571.233.

^1^H-NMR (950 MHz, H_2_O/D_2_O (9:1, v/v)), δ (ppm): 1.986, 2.069 (2H, m, m, C^β^H_2_ Met^5^), 2.496, 2.576 (1H, m, m, C^γ^H Met^5^), 2.765, 3.373 (2H, dd, dd, C^β^H_2_ Phe^4^), 3.075, 3.221 (2H, dd, dd, C^β^H_2_ Tyr^1^), 3.538, 4.082 (2H, dd, m?, C^α^H_2_ Gly^2^), 3.609, 3.694 (2H, dd, dd, C^α^H_2_ Gly^3^), 4.091 (1H, m, C^α^H Tyr^1^), 4.442 (1H, m, C^α^H Met^5^), 4.741 (1H, C^α^H Phe^4^), 6.904 (1H, d, C^ε^H Tyr^1^), 7.093, 7.323 (2H, d, s, C^δ^H Tyr^1^), 7.199 (2H, d, C^δ^H Phe^4^), 7.317 (1H, d, C^ε^H Phe^4^), 7.807 (1H, t, NH Gly^3^), 7.908 (1H, d, NH Phe^4^), 8.183 (1H, broad s?, NH Gly^2^), 8.481 (1H, d, NH Met^5^), n.a. (1H, NH Tyr^1^).

Linear peptides

**Leu-enkephalin - H-Tyr-Gly-Gly-Phe-Leu-OH.** Peptide was synthesized according to General Procedure A on Fmoc-Leu-Wang resin (0.71 mmol/g).

HPLC R_t_: 18.8 min, purity: 100%, yield 71%. ESI-MS: *m/z* ([M+H]^+^) found 556.277, calcd. for C_28_H_37_N_5_O_7_ 556.277.

**Leu-enkephalin amide - H-Tyr-Gly-Gly-Phe-Leu-NH_2_.** Peptide was synthesized according to General Procedure A on Fmoc-Rink Amide MBHA resin (0.68 mmol/g).

HPLC R_t_: 17.73 min, purity: 99.3%, yield 79%. ESI-MS: *m/z* ([M+H]^+^) found 555.295, calcd. for C_28_H_38_N_6_O_6_ 555.292.

**Met-enkephalin amide - H-Tyr-Gly-Gly-Phe-Met-NH_2_.** Peptide was synthesized according to General Procedure A on Fmoc-Rink Amide MBHA resin (0.68 mmol/g).

HPLC R_t_: 15.66 min, purity: 94.8%, yield 68%. SI-MS: *m/z* ([M+H]^+^) found 573.251, calcd. for C_27_H_36_N_6_O_6_S 573.249.

**H-Tyr-Gly-Gly-Tyr-Leu-NH_2_.** Peptide was synthesized according to General Procedure A on Fmoc-Rink Amide MBHA resin (0.68 mmol/g).

HPLC R_t_: 14.60 min, purity: 97.8%, yield 68%. ESI-MS: *m/z* ([M+H]^+^) found 571.290, calcd. for C_28_H_38_N_6_O_7_ 571.287.

**H-Phe-Gly-Gly-Phe-Leu-NH_2_.** Peptide was synthesized according to General Procedure A on Fmoc-Rink Amide MBHA resin (0.68 mmol/g).

HPLC R_t_: 19.82 min, purity: 99.4%, yield 76%. ESI-MS: *m/z* ([M+H]^+^) found 539.301, calcd. for C_28_H_38_N_6_O_5_ 539.298.

**H-Phe-Gly-Gly-Tyr-Leu-NH_2_.** Peptide was synthesized according to General Procedure A on Fmoc-Rink Amide MBHA resin (0.68 mmol/g).

HPLC R_t_: 16.56 min, purity: 100%, yield 75%, ESI-MS: *m/z* ([M+H]^+^ found 555.291, calcd. for C_28_H_38_N_6_O_6_ 555.292.

**Table S-4.** Parameters of NMR measurements of the peptides studied.

**Table S-4 (contd.).**

**Listing S-1.** Atomic coordinates of **c-(Tyr-*m*-Phe-*p*)-M-NH_2_** molecule, lowest-energy geometry calculated by XPLOR. Starting point for QM optimization.

N 6.70000000 -3.52900000 1.36400000

H 6.45600000 -3.76100000 0.38100000

H 6.44600000 -4.32600000 1.98300000

H 7.72200000 -3.34900000 1.43200000

C 5.95100000 -2.31200000 1.78900000

H 5.90700000 -2.27700000 2.86300000

C 6.65800000 -1.05700000 1.24500000

H 6.28500000 -0.84700000 0.23400000

H 7.73200000 -1.23700000 1.21700000

C 6.39700000 0.14900000 2.12800000

C 6.03200000 1.37300000 1.55100000

H 5.90300000 1.44100000 0.48800000

C 6.55200000 0.06000000 3.50500000

H 6.81700000 -0.87600000 3.96300000

C 5.82000000 2.51500000 2.34400000

C 6.34300000 1.17900000 4.29700000

H 6.46000000 1.10600000 5.34900000

C 5.98800000 2.39800000 3.72700000

O 5.79600000 3.49900000 4.53700000

H 6.61300000 3.66300000 5.01400000

C 4.54300000 -2.37100000 1.22800000

O 3.96400000 -3.44200000 1.04900000

N 4.01700000 -1.19600000 0.94100000

H 4.54600000 -0.38700000 1.10200000

C 2.69000000 -1.06300000 0.38100000

H 2.35100000 -1.99400000 -0.03600000

H 2.72200000 -0.30400000 -0.39600000

C 1.70100000 -0.61000000 1.40400000

O 0.49500000 -0.73000000 1.18600000

N 2.17700000 0.00700000 2.48000000

H 3.13900000 0.15700000 2.59100000

C 1.22600000 0.56100000 3.40800000

H 1.72900000 1.19900000 4.13900000

H 0.66000000 -0.24400000 3.88700000

C 0.34200000 1.40700000 2.50700000

O -0.70600000 1.93300000 2.88200000

N 0.86400000 1.49300000 1.27200000

H 1.71000000 1.03100000 1.10700000

C 0.28600000 2.21900000 0.16600000

H -0.78500000 2.28700000 0.27900000

C 0.89300000 3.60700000 0.13700000

H 0.28500000 4.24900000 0.73500000

H 0.95900000 3.96100000 -0.89600000

C 2.26800000 3.55600000 0.73900000

C 2.40000000 3.47400000 2.11800000

H 1.52500000 3.46600000 2.74300000

C 3.40200000 3.57100000 -0.06900000

H 3.30500000 3.64800000 -1.14100000

C 3.65500000 3.39200000 2.69700000

H 3.73400000 3.33100000 3.77100000

C 4.66900000 3.49600000 0.51000000

H 5.54400000 3.50200000 -0.12200000

C 4.81600000 3.38900000 1.90600000

C 0.63900000 1.51800000 -1.14700000

O -0.22800000 0.99600000 -1.84800000

N 1.93600000 1.53100000 -1.46200000

H 2.56600000 1.97200000 -0.85700000

C 2.44500000 0.91900000 -2.68600000

H 1.95100000 1.37600000 -3.53000000

C 3.95800000 1.17600000 -2.81000000

H 4.14100000 2.25600000 -2.76300000

H 4.30800000 0.77300000 -3.76700000

C 4.73000000 0.48500000 -1.66800000

H 4.48700000 -0.56700000 -1.65400000

H 4.47100000 0.93300000 -0.70800000

S 6.50600000 0.68000000 -1.96000000

C 6.73900000 -0.81600000 -2.95100000

H 6.85700000 -1.66700000 -2.29400000

H 7.62200000 -0.71200000 -3.56100000

H 5.87800000 -0.96300000 -3.58900000

C 2.16400000 -0.58200000 -2.71400000

O 1.19300000 -1.04700000 -2.11700000

N 2.96300000 -1.37200000 -3.37700000

H 3.73500000 -1.00100000 -3.85200000

H 2.79000000 -2.33600000 -3.40100000

**Listing S-2.** Atomic coordinates of **c-(Tyr-*m*-Phe-*p*)-M-NH**_2_ molecule optimized at the B3LYP/6-31G(d,p) level with PCM solvent model (water). The minimum found starting from the lowest-energy structure calculated by XPLOR.

Sum of electronic and thermal Free Energies= -2227.482870

0 imaginary frequencies

Charge = 1 Multiplicity = 1

N 4.41446 -4.0399 1.64764

H 3.56709 -4.61815 1.55907

H 4.64682 -3.9766 2.64344

H 5.19306 -4.51127 1.17893

C 4.11531 -2.68455 1.0377

H 4.89945 -2.00277 1.36905

C 4.11803 -2.84764 -0.49994

H 3.24893 -3.45584 -0.77375

H 5.01203 -3.41806 -0.77224

C 4.125 -1.53928 -1.2589

C 2.9547 -0.79109 -1.415

H 2.02623 -1.15681 -0.98627

C 5.30783 -1.03851 -1.81405

H 6.23488 -1.5958 -1.71395

C 2.92896 0.44385 -2.0777

C 5.31133 0.17054 -2.50545

H 6.2321 0.54073 -2.94888

C 4.13568 0.91648 -2.63677

O 4.10857 2.10315 -3.30984

H 4.99723 2.31092 -3.63215

C 2.71704 -2.34542 1.60222

O 1.8481 -3.21466 1.52447

N 2.53445 -1.13436 2.17202

H 3.35245 -0.57004 2.36505

C 1.31772 -0.87833 2.94661

H 1.38583 -1.34421 3.93394

H 0.47563 -1.33369 2.41457

C 0.99202 0.59387 3.19276

O 0.47984 0.93315 4.25597

N 1.22919 1.4662 2.17151

H 1.68379 1.12871 1.33326

C 1.01242 2.89661 2.32094

H 1.8588 3.44654 1.90321

H 0.95832 3.11892 3.38943

C -0.25286 3.47078 1.66182

O -0.40418 4.68901 1.57524

N -1.15067 2.56228 1.21617

H -0.88857 1.58973 1.29938

C -2.33605 2.91884 0.44104

H -2.76259 3.81582 0.89067

C -2.0355 3.24157 -1.07931

H -1.93175 4.32631 -1.1624

H -2.91029 2.95651 -1.6722

C -0.77846 2.57982 -1.58973

C 0.45289 3.23842 -1.45842

H 0.47039 4.26468 -1.10343

C -0.77005 1.2677 -2.0803

H -1.70969 0.75369 -2.26163

C 1.65422 2.5798 -1.70187

H 2.5927 3.10166 -1.55315

C 0.4315 0.59788 -2.31299

H 0.41085 -0.43071 -2.66072

C 1.66557 1.22611 -2.07942

C -3.40482 1.83169 0.63544

O -4.5359 2.11461 1.02606

N -3.01798 0.55664 0.36146

H -2.05611 0.34841 0.11551

C -3.76127 -0.59792 0.84075

H -4.37701 -0.26728 1.68401

C -4.71717 -1.19673 -0.22123

H -5.41078 -0.40341 -0.51485

H -5.31455 -1.98219 0.25336

C -4.00634 -1.7493 -1.45834

H -3.31372 -2.55616 -1.19755

H -3.42529 -0.96294 -1.94803

S -5.1375 -2.36448 -2.76777

C -5.78702 -3.89205 -1.99823

H -4.97101 -4.5705 -1.73875

H -6.42339 -4.37032 -2.74541

H -6.38712 -3.67451 -1.11272

C -2.71639 -1.6108 1.35097

O -1.51428 -1.4549 1.11041

N -3.20055 -2.66099 2.03862

H -4.17435 -2.74442 2.28732

H -2.55573 -3.35116 2.39626

**Table S-5.** Docking scores for top MOR docking poses of **[Met^5^]enk-NH_2_**_._

| **Pose No** | **AutoDock Vina docking score [kcal/mol]** | **Comments** |
| --- | --- | --- |
| **1** | -8.6 | Taken for further analyses. |
| **2** | -8.3 | The canonical interaction lacking. |
| **3** | -8.3 | The canonical interaction lacking. |
| **4** | -8.2 | The canonical interaction lacking. |
| **5** | -8.2 | The canonical interaction lacking. |
| **6** | -8.2 | The canonical interaction lacking. |
| **7** | -8.1 | The canonical interaction lacking. |
| **8** | -8.1 |  |
| **9** | -8.1 | The canonical interaction lacking. |

**Table S-6.** Docking scores for top MOR docking poses of **c-(Tyr-*m*-Phe-*p*)-M-NH_2_**.

| **Pose No** | **AutoDock Vina docking score [kcal/mol]** | **Comments** |
| --- | --- | --- |
| **1** | -8.2 | The one taken for further analysis. |
| **2** | -8.1 | - |
| **3** | -8.1 | **-** |
| **4** | -8.0 | - |
| **5** | -8.0 | - |
| **6** | -7.8 | - |
| **7** | -7.8 | - |
| **8** | -7.8 | - |
| **9** | -7.8 | - |
